# A multi-functional oral small molecule targeting energy and lipid metabolism to treat obesity and related metabolic disorders

**DOI:** 10.1126/sciadv.aed3119

**Published:** 2026-08-21

**Authors:** Justin Y. Lee, Chi Zhu, Melissa A. Boldridge, Rachelle L. Stark, Gracia Bonilla, Kosuke Watari, Christina Papa, Lei Xu, Federico Gonzalez, Xin Tang, Kaitlyn T. Dang, Kook Son, Kashish Chetal, Prabha Ibrahim, Ruslan I. Sadreyev, Bilal N. Sheikh, Michael Karin, Anders M. Näär

**Affiliations:** ^1^ Department of Nutritional Sciences & Toxicology, University of California, Berkeley, CA 94720, USA.; ^2^ Department of Molecular Biology, Massachusetts General Hospital, Boston, MA 02114, USA.; ^3^ Department of Pharmacology, School of Medicine, University of California, San Diego, La Jolla, CA 92093, USA.; ^4^ Helmholtz Institute for Metabolic, Obesity and Vascular Research (HI-MAG) of the Helmholtz Center Munich, 04103 Leipzig, Germany.; ^5^ ReRx Therapeutics, Inc., Berkeley, CA 94704, USA.; ^6^ Department of Pathology, Massachusetts General Hospital and Harvard Medical School, Boston MA 02114, USA.

## Abstract

Obesity and related metabolic disorders have surged globally, and are mechanistically interconnected through dysregulated energy and lipid metabolism pathways. Current incretin-based obesity treatments act to decrease food intake, but are associated with gastrointestinal side effects and muscle wasting. Here, we identified an orally bioavailable multi-functional small molecule, 5-tetradecyloxy-2-furoic acid (TOFA), that promotes energy expenditure and rebalances lipid synthesis, thereby significantly alleviating obesity, abnormal glucose homeostasis and fatty liver-related diseases without affecting food intake or muscle mass. Mechanistically, TOFA inhibits the lipogenic enzymes acetyl-CoA carboxylases 1 and 2 (ACC1/2) and activates the Peroxisome Proliferator-Activated Receptors alpha and delta (PPARα/δ), key regulators of energy expenditure and lipid metabolism gene expression programs. TOFA acted more than additively with incretin analogs such as semaglutide and tirzepatide to improve obesity, dyslipidemia, and insulin resistance. Our findings suggest that the coordinated multi-targeting of energy metabolism and lipid homeostasis by TOFA is an effective approach to address multiple associated metabolic diseases.

## INTRODUCTION

Obesity remains a global public health burden (World Obesity Federation; worldobesity.org). Associated metabolic disorders such as dyslipidemias, metabolic syndrome, insulin resistance, diabetes mellitus, and metabolic dysfunction-associated steatotic liver disease (MASLD)/metabolic dysfunction-associated steatohepatitis (MASH) negatively impact quality of life and are projected to become leading causes of morbidity and mortality (*1*, *2*). Traditional interventions such as diet modifications and physical activity can be effective, but are often insufficient for sustained body-weight loss (*3*–*5*). Current marketed pharmacological products and investigational efforts are primarily focused on decreased energy intake, as emphasized by the blockbuster incretin analog drugs that suppress appetite and increase satiety (*6*, *7*). Although recent approvals of injectable and oral incretin-based therapeutics for obesity and diabetes treatment represent a notablemilestone in addressing these prevalent metabolic diseases, side effects such as loss of muscle mass and gastrointestinal symptoms limit the impact and treatment adherence. Hence, there is strong interest in developing therapeutic avenues that can more comprehensively address metabolic disease manifestations.

Dysregulation of energy homeostasis is a hallmark of metabolic disease progression associated with obesity, and is strongly linked to abnormal lipid metabolic regulation, especially as it pertains to fatty acid synthesis and breakdown (*8*). To effectively address and target processes related to lipid imbalances and dysregulated metabolic homeostasis, various strategies and target classes have been pursued for therapeutic applications (*9*, *10*). Among potential targets in lipid and energy metabolism, acetyl-CoA carboxylase 1 and 2 (ACC1/2) are appealing due to their nonredundant roles as rate-limiting enzymes in the de novo lipogenesis (DNL) pathway and regulators of fatty acid beta-oxidation (*11*–*17*). Previous studies in mice and in human clinical trials have also demonstrated the therapeutic potential of ACC1/2 inhibition in addressing MASLD and fibrotic MASH (*18*–*21*). However, safety concerns over ACC inhibition arise from treatment-induced hypertriglyceridemia, a serious adverse metabolic side effect hypothesized to stem from compensatory upregulation of LXR and SREBP1c from decreases in polyunsaturated fatty acids (PUFAs) (*18*, *22*, *23*). The reported increase in blood lipid levels has been consistently seen with various ACC inhibitors, genetic *Acc* knockout mouse models, and in human clinical trials, making hypertriglyceridemia-mediated predisposition to cardiovascular disease a class effect and a substantial shortcoming of ACC inhibition as a monotherapy.

Early studies characterized 5-tetradecyloxy-2-furoic acid (TOFA) as an orally bioavailable small molecule inhibitor of ACC1/2 that lowers blood lipids, presenting an unresolved discrepancy of an ACC inhibitor without its known class effect of hyperlipidemia (*24*, *25*). We speculated TOFA may work through additional activities to confer its lipid-lowering effects. TOFA has demonstrated favorable tolerability (LD_50_: >5 g/kg in rats) and efficacy signals in rats (*26*–*28*), hamsters (*29*), and monkeys (*30*). However, TOFA was never evaluated for broader metabolic effects in the context of obesity, metabolic syndrome, insulin resistance/type 2 diabetes, and MASLD/MASH, and furthermore has not been evaluated in humans. In the current studies, we have carried out comprehensive studies of the effect of TOFA in various metabolic disease models pertaining to obesity and associated comorbidities. Notably, orally delivered TOFA exhibited a dramatic beneficial effect in treating high-fat diet-induced obesity in mice, without the adverse elevation of blood triglycerides as ordinarily seen with other ACC inhibitors. Obesity comorbidities such as diabetes-related insulin resistance and glucose intolerance, as well as MASLD/MASH phenotypes (hepatic steatosis, hepatocyte ballooning, inflammation and fibrosis) were also markedly ameliorated. Importantly, orally delivered TOFA also acts additively or synergistically with injectable incretin analogs, such as semaglutide and tirzepatide, in countering diet-induced obesity and its comorbidities. Our mechanistic studies revealed that TOFA not only inhibits ACC1/2 to prevent lipid production and stimulate fatty acid beta-oxidation, but also activates the Peroxisome Proliferator-Activated Receptors alpha and delta (PPARα and PPARδ) nuclear hormone receptors, key transcriptional regulators of lipid homeostasis and energy expenditure. Taken together, our studies have identified TOFA as a potent, specific multi-functional regulator of lipid and energy metabolism that boosts energy expenditure, providing the rationale for further clinical consideration of TOFA to reduce obesity and associated comorbidities, alone or in conjunction with incretin analogs.

## RESULTS

### TOFA is a unique orally bioavailable ACC1/2 inhibitor

**Fig. 1.**
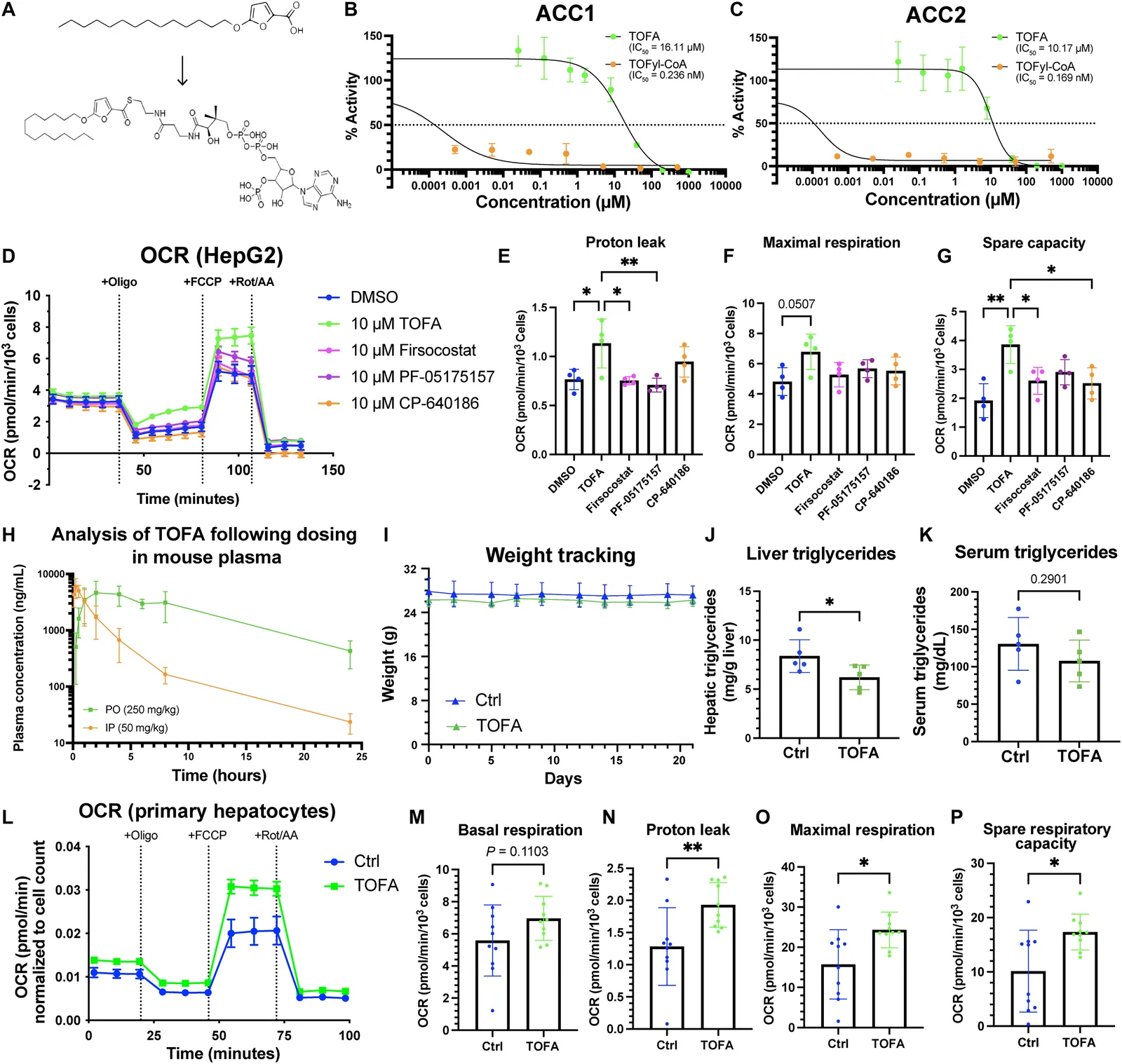
Dual ACC inhibitor TOFA exhibits unique structural and functional properties in vitro and is orally bioavailable and tolerated in vivo, both demonstrating differentiated functional outcomes compared to other ACC candidates. (**A**) Chemical structure of TOFA and intracellular TOFyl-CoA. (**B**) Inhibition of ACC1 activity by TOFA or TOFyl-CoA. (**C**) Inhibition of ACC2 activity by TOFA or TOFyl-CoA. (**D** to **G**) HepG2 cells were seeded in the Seahorse XF plate, treated with 10 μM of various ACC inhibitors (maximal inhibitory dose; IC_50_ < 10 μM), and processed through the mitochondrial stress test. (D) Oxygen consumption rate plotted during treatment time course; *n* = 4/group. Subsequently, calculation of various mitochondrial respiration parameters: (E) proton leak; *P = 0.0191, *P = 0.0151, **P = 0.0064, (F) maximal respiration, and (G) spare respiratory capacity; **P = 0.0011, *P = 0.0358, *P = 0.0229. (**H**) Mice (*n* = 3/group) were administered a single dose of TOFA (50 mg/kg and 250 mg/kg via IP and PO, respectively) and TOFA concentration was measured in plasma samples at various indicated collection timepoints. (**I** to **K**) Eleven-week-old male, chow-fed C57BL/6 J mice had vehicle or TOFA administered orally (125 mg/kg, BID) for three weeks; *n* = 5/group; (I) Body weight over treatment time course. (J) Liver triglycerides; *P = 0.0491. (K) Serum triglycerides. (**L**) Male C57BL/6 J mice were fed chow, then vehicle or TOFA was administered orally (250 mg/kg/day) for one week. Mice were euthanized, primary hepatocytes were isolated, and processed through the mitochondrial stress test for OCR and plotted; *n* = 10 replicates. Subsequently, calculation of various mitochondrial respiration parameters: (**M**) basal respiration; *P* = 0.1103, (**N**) proton leak; **P = 0.0087, (**O**) maximal respiration; *P = 0.0118, and (**P**) spare respiratory capacity; *P = 0.0128. Data are presented as mean ± SD. *P < 0.05, **P < 0.01 (unpaired two-tailed *t* test).

Previous studies have defined TOFA as a low micromolar inhibitor of fatty acid and cholesterol synthesis in hepatocytes at the level of ACC1 and ACC2 (*31*). Given TOFA’s unique structural properties that share similarities with fatty acids, TOFA has been shown to similarly be converted intracellularly into its thioester form, TOFyl-CoA, which represents a more potent ACC inhibitor than the parent TOFA compound (*24*, *32*, *33*) (Fig. 1A). As expected, our biochemical profiling of the TOFyl-CoA thioester form demonstrated markedly stronger inhibition of both ACC1 and ACC2 as compared to the free form TOFA (Fig. 1, B and C). TOFA treatment of HepG2 hepatoma cells, compared to other characterized ACC inhibitors, such as Firsocostat (*19*), PF-05175157 (*20*), and CP-640186 (*34*), uniquely resulted in higher oxygen consumption rates (Fig. 1D) via increased proton leak (Fig. 1E) and elevated spare mitochondrial capacity for ATP production during metabolic stress or demand (Fig. 1, F and G and fig. S1, A to C), suggesting TOFA’s distinct mechanism beyond just ACC inhibition in enhancing cellular energy expenditure. Moreover, among lipogenic enzyme modulators more broadly, TOFA exhibited a distinctly elevated mitochondrial capacity profile, especially with proton leak and spare respiratory capacity, further highlighting the distinct mechanism of TOFA as a lipid and energy metabolism modulating agent (fig. S1, D and E). To assess in vivo tolerability of TOFA, clearance of TOFA in C57BL/6 J mice following oral administration showed longer half-life clearance following oral administration (PO) compared to intraperitoneal administration (IP) (Fig. 1H), providing justification for subsequent oral administration of TOFA. In healthy, chow-fed C57BL/6 J mice orally administered with TOFA for three weeks, no significant change in weight was observed compared to vehicle treated mice (Fig. 1I). Interestingly, TOFA treatment reduced liver triglyceride levels (Fig. 1J), without an increase in serum triglyceride levels, a noted adverse class side effect of ACC inhibition (Fig. 1K). To further understand the underlying hepatic effects of TOFA in vivo, primary hepatocytes were isolated from C57BL/6 J mice treated once daily by oral gavage for one week with either vehicle or TOFA, and subsequently evaluated by mitochondrial stress testing. Hepatocytes from TOFA-treated mice exhibited higher oxygen consumption rates throughout the mitochondrial stress test, indicating higher mitochondrial respiratory function in response to TOFA treatment in mice, consistent with our in vitro findings (Fig. 1, L to P). Together, our data demonstrate a differentiated mechanism of action and outcome of TOFA compared to similar classes of lipid modulating compounds and supports TOFA’s bioavailability, efficacy, and tolerability in mice.

### TOFA treatment counters metabolic derangements in a high-fat diet-induced mouse model of obesity and metabolic syndrome

**Fig. 2.**
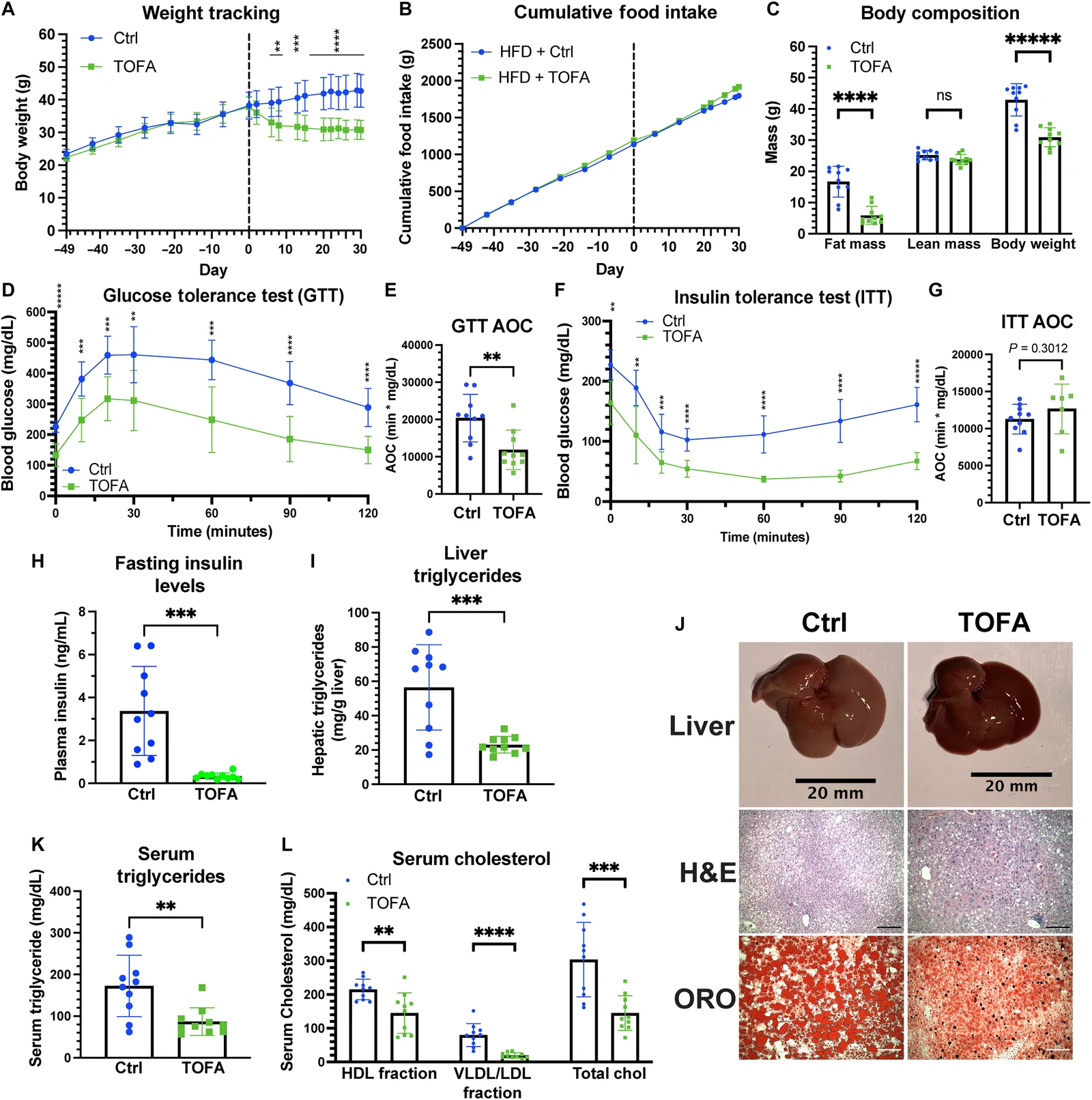
Oral TOFA treatment resolves diet-induced obesity metabolic dysregulation. [(A) to (L)] Male C57BL/6 J mice were placed on a 60% HFD for seven weeks and treated for four weeks with vehicle or TOFA (125 mg/kg, BID loading dose for one week followed by 62.5 mg/kg, BID maintenance dose); *n* = 10/group. (**A**) Body weight; **P < 0.01, ***P < 0.001, ****P < 0.0001. (**B**) Food intake. (**C**) EchoMRI body composition analysis; ****P = 1.099E-05, *P* = 0.053 (ns), *****P = 6.2E-06. (**D**) Glucose tolerance test. (**E**) Area of the curve (AOC) from glucose tolerance test (GTT); **P = 0.0043. (**F**) Insulin tolerance test. (**G**) Area of the curve (AOC) from insulin tolerance test (ITT); *P* = 0.3012. (**H**) Fasting blood insulin levels; ***P = 0.0002. (**I**) Liver triglycerides; ***P = 0.0006. (**J**) Representative whole liver, H&E, Oil Red O (ORO) staining of liver sections. Scale bars, 20 mm or 200 μM (inset). (**K**) Serum triglycerides; **P = 0.0035. (**L**) Serum total, HDL, and VLDL/LDL cholesterol fractions; **P = 0.004, ****P = 0.0000351, ***P = 0.000676. Data are presented as mean ± SD. ns, not significant; *P < 0.05, **P < 0.01, ***P < 0.001, ****P < 0.0001 (unpaired two-tailed *t* test).

Given the effects of TOFA on mitochondrial energy expenditure in hepatic cells, we wished to assess whether TOFA might also boost energy expenditure in vivo and beneficially affect obesity and related metabolic disorders. Male C57BL/6 J mice were fed a 60 kcal% fat diet (HFD) for seven weeks to induce rapid weight gain and diet-induced obesity (DIO), then orally treated twice-daily for four weeks with vehicle or TOFA. Body weight reduction was immediately observed after TOFA introduction and sustained after switching to a lower maintenance dose (to prevent rapid weight loss exceeding defined humane endpoints), equating to an average 18% decrease in body weight at the end of the treatment course (Fig. 2A). The effect on weight change was dose-dependent, in which the initiation dose conferred the strongest effects on body weight change (fig. S2A). Treatment caused no changes in food intake (Fig. 2B), and weight loss was primarily attributed to a significant fat mass reduction with no significant changes in lean mass as assessed by EchoMRI (Fig. 2C). Weight loss is known to improve type 2 diabetes–related phenotypes such as insulin resistance and glucose intolerance in obese mammals. TOFA treatment significantly lowered baseline fasting blood glucose and improved total glucose tolerance observed by earlier blood glucose level recovery response initiation starting at thirty minutes after glucose injection (Fig. 2, D and E), a concerted effect of both weight loss and prolonged insulin sensitivity (Fig. 2, F and G and fig. S3, A and B). TOFA treatment also reduced fasting insulin levels (Fig. 2H), an anti-diabetic effect. Obesity is also strongly associated with fatty liver diseases (e.g., MASLD). Strikingly, TOFA treatment significantly reduced liver triglycerides (Fig. 2I) and visibly improved liver morphology with less lipid accumulation (Fig. 2J), indicating protective effects of TOFA against hepatic steatosis. These results align with the dose-dependent efficacy of TOFA in modulating liver lipid levels (fig. S2B). We also observed a significant decrease in various circulating lipid species, including serum triglycerides (Fig. 2K) and cholesterol, in which the reduction was more pronounced in the VLDL/LDL cholesterol fraction over the HDL cholesterol fraction (Fig. 2L), consistent with beneficial remodeling of the blood lipid profile. Changes in blood lipid levels were also observed in a dose-dependent manner with TOFA treatment (fig. S2, C and D). These results present TOFA as an ACC1/2 inhibitor with a unique mechanism of action that addresses broader metabolic risk factors and complications associated with diet-induced obesity.

### TOFA treatment confers metabolic and energy homeostasis benefits against diet-induced obesity

**Fig. 3.**
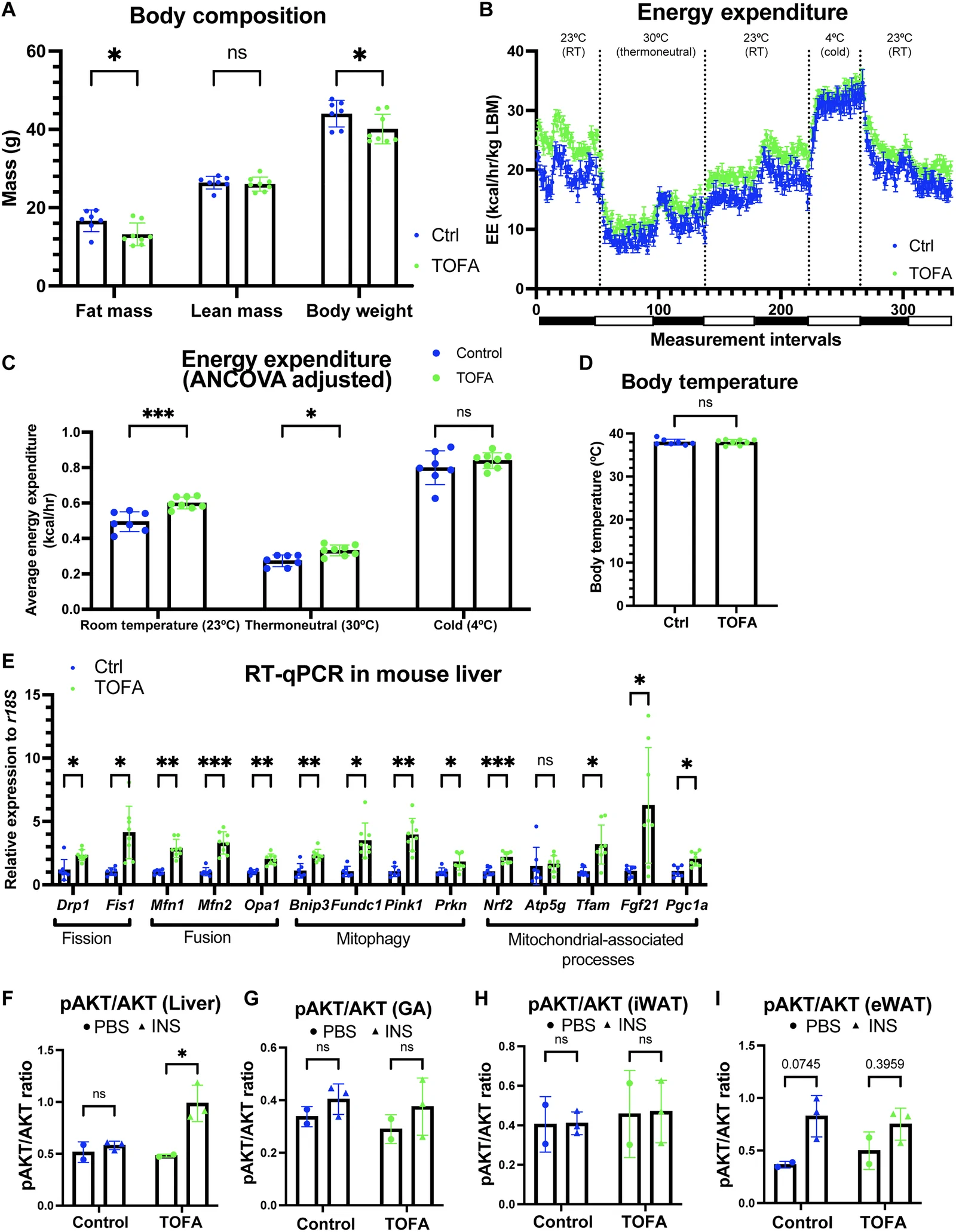
TOFA reduces body mass and promotes energy expenditure and restores metabolic homeostasis in mice. (**A** to **I**) Male C57BL/6 J mice were placed on a 60% HFD for twelve weeks and treated for one week with vehicle or TOFA (200 mg/kg/day), OD, before metabolic cage analysis. After metabolic cage analysis, treatment resumed for an additional three weeks; *n* = 7–8/group. (A) EchoMRI body composition analysis at one week right before metabolic cage analysis; *P = 0.0228, *P* = 0.8 (ns), and *P = 0.0107 (two-way ANOVA). (B) CLAMS metabolic cage analysis performed when body weight between groups reached significance (*P* < 0.05). (C) Average energy expenditure adjusted for lean mass and fat mass as covariates using ANCOVA analysis (*35*); ***P = 0.0004, *P = 0.0379, *P* = 0.1462 (ns) (two-way ANOVA). (D) Body temperature; *P* = 0.971 (ns). (E) RT-qPCR in mouse liver of mitochondrial function genes. (F to I) Ten minutes prior to tissue collection, mice were injected with either PBS or Insulin (1 U/kg) and phosphorylated AKT and total AKT ratio was assessed by ELISA assay in (F) liver, (G) gastrocnemius muscle (GA), (H) inguinal adipose tissue (iWAT), or (I) brown adipose tissue (BAT), respectively. Data are presented as mean ± SD. ns, not significant; *P < 0.05, **P < 0.01, ***P < 0.001, ****P < 0.0001 (unpaired two-tailed *t* test, unless otherwise indicated).

To gain a better mechanistic understanding of how TOFA confers its metabolic benefits beyond lipid regulation, energy expenditure was assessed in vivo. In order to investigate the dynamic period of weight change (as opposed to assessing treatment end outcomes), C57BL/6 J mice were fed a 60% HFD for twelve weeks followed by a short one-week course of vehicle or TOFA once daily oral treatment. At the one-week timepoint, TOFA-treated mice started to show significant body weight change compared to vehicle treated mice, with a specific reduction in fat mass and no significant changes in lean mass (Fig. 3A). Gross fecal energy content revealed no caloric differences between control and TOFA-treated mice, indicating no signs of malabsorption and the energy phenotype driven by internal cellular metabolism rather than gastrointestinal losses (fig. S4A). Mice were subsequently placed in metabolic cages. Interestingly, TOFA treated mice exhibited increased energy expenditure at all housing temperature conditions, ranging from room temperature (23°C), thermoneutral (30°C), and less pronounced in cold exposure (4°C) (Fig. 3B and fig. S4B), suggesting broad stimulation of energy expenditure beyond low temperature-dependent thermogenesis mediated by brown and beige adipose tissues. To account for differences in body composition proportions potentially contributing to metabolic energy expenditure, ANCOVA adjustment of energy expenditure (*35*) was carried out, which further confirmed our observation that TOFA treatment increases energy expenditure through non-thermogenic mechanisms, with significant contribution by lean mass (fig. S4, C and E and table S1). Indeed, we observed ∼18% increase in energy expenditure in TOFA-treated mice compared to control under both room temperature and thermoneutral conditions, in which thermogenic demands are minimal, whereas energy expenditure increase was minimal under cold exposure (4% increase in TOFA-treated mice), supporting the notion of genuine metabolic activation by TOFA rather than enhanced thermogenesis mechanisms (Fig. 3C). This increase in overall energy expenditure was consistent with the subcomponent increases in oxygen consumption (fig. S4, F and G) and carbon dioxide production (fig. S4, H and I), which reveal fat oxidation as the predominant energy source (fig. S4, J and K). Furthermore, the increase in energy expenditure is not attributed to significant increases in overall activity levels (fig. S4, L and M), suggesting whole body metabolic thermogenesis. Measurement of body temperature revealed no evidence of hyperthermia, an adverse effect of concern for energy metabolism modulating agents (Fig. 3D). The modest rise in energy expenditure in response to TOFA treatment alleviates concerns related to increased cardiac workload demand, as previous studies support the tolerability of 10–20% increases in energy expenditure (*36*, *37*). The boost in energy expenditure by TOFA in vivo also aligns with the increased mitochondrial oxygen consumption rate observed from TOFA treatment in both human hepatocellular cell lines (Fig. 1D) and primary murine hepatocytes (Fig. 1L). In agreement with these findings, gene expression analysis with RT-qPCR of livers from TOFA-treated animals revealed a marked upregulation of genes involved in mitochondrial fusion, fission, mitophagy, and other mitochondria-associated bioenergetic processes, suggesting broad beneficial mitochondrial dynamic remodeling in response to TOFA treatment (Fig. 3E). Given the critical role of mitochondria in energy production and oxidative stress regulation, the impact of TOFA on mitochondrial dynamics for maintaining bioenergetic function, restoring cellular health, and adapting to metabolic needs is highly significant. Another aspect of metabolic benefits conferred by TOFA is the improvements in insulin resistance, an obesity-related hallmark of metabolic syndrome and pre-diabetes. Before tissue collection, mice were injected with PBS or insulin (1 U/kg) to assess insulin signaling via the pAKT to AKT ratio. As expected, control DIO mice were insulin resistant in multiple tissues, showing no change in pAKT levels between PBS and insulin treatment (Fig. 3, F to I). However, TOFA treatment robustly restored liver insulin sensitivity, as evidenced by a significant increase in the pAKT to AKT ratio after insulin injection (Fig. 3F). Given the remarkable beneficial metabolic effects in obese mice, we speculated that TOFA might also confer extrahepatic metabolic and energetic benefits. Of note, in an endurance exercise treadmill test, no decrease in endurance muscle function was observed (fig. S5A), consistent with our observation of preservation of lean mass with TOFA treatment. Interestingly, examination of gastrocnemius muscle, a mixed fiber type muscle, revealed upregulation of genes involved in the uncoupling response and mitochondrial biogenesis, suggesting peripheral, extrahepatic bioenergetic effects of TOFA (fig. S5B). Another potential contributor to elevated energy expenditure with TOFA is stimulation of brown adipose tissue (BAT) or the beigeing (or browning) of subcutaneous white adipose depots. However, canonical genes involved in BAT function and WAT browning (e.g., *Pgc1a*, *Prdm16*, *Ucp1*) showed only marginal and inconsistent increases, suggesting these processes are unlikely to explain the robust effect of TOFA on whole-body energy expenditure (fig. S5, C and D). Moreover, the stronger elevation in energy expenditure at room temperature and thermoneutral conditions as compared with cold exposure (Fig. 3C) further support a broad increase of systemic metabolism beyond classical brown or beige adipose thermogenesis. Taken together, these findings highlight the role of TOFA in modulating energy and metabolic homeostasis in various tissue depots, likely contributing to its potent anti-obesity effect.

### TOFA treatment has a distinct transcriptional profile revealing upregulation of the PPAR transcriptional network

**Fig. 4.**
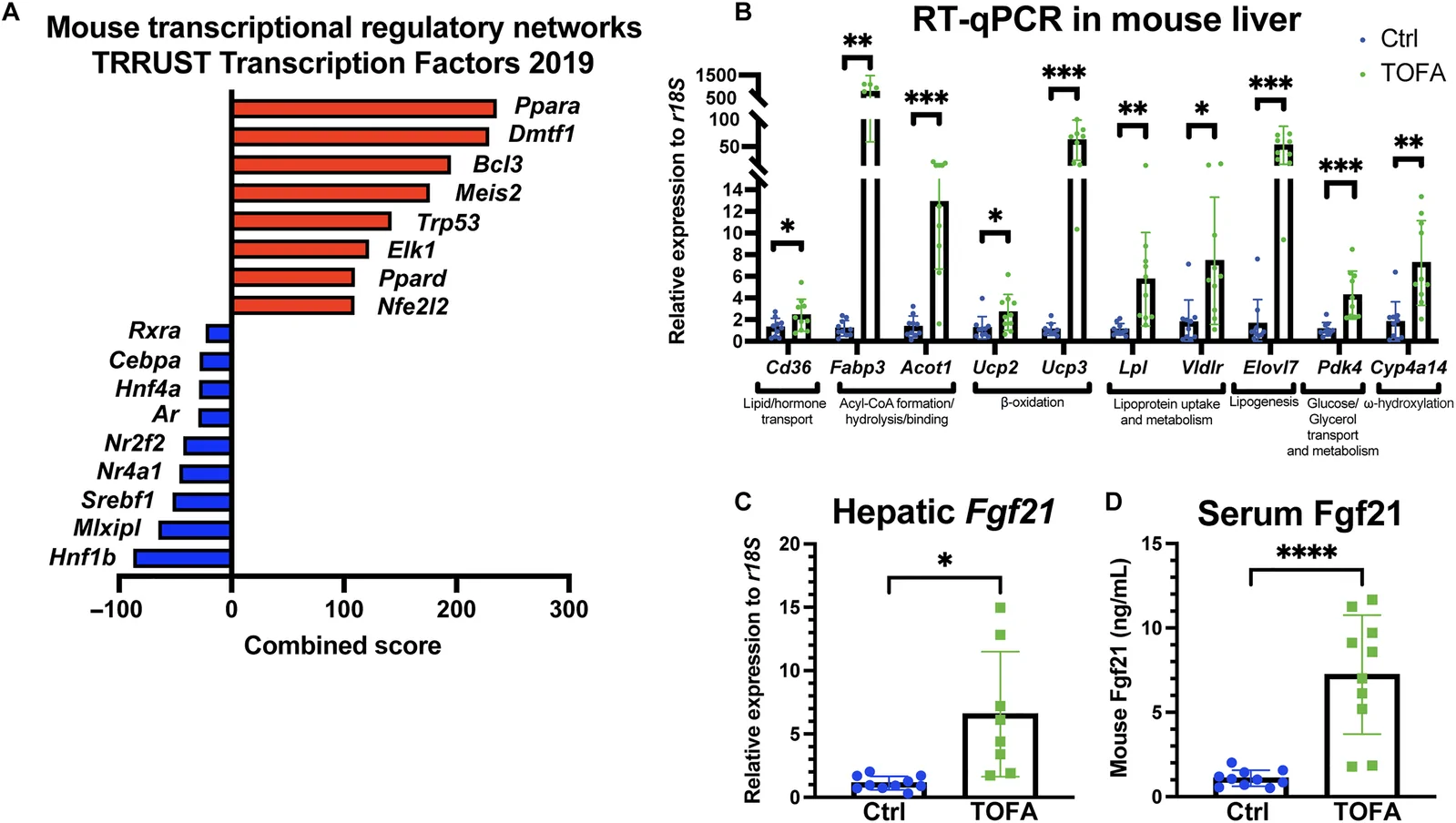
RNA-sequencing revealed a PPAR transcriptional network signature by TOFA treatment. Liver tissue of male C57BL/6 J mice placed on a 60% HFD for seven weeks and treated for four weeks with vehicle or TOFA (125 mg/kg, BID loading dose for one week followed by 62.5 mg/kg, BID maintenance dose), as above, were isolated, extracted for RNA, and subjected to RNA sequencing; *n* = 10/group (from Fig. 2). (**A**) Top upregulated (combined score > 100 and *P* < 0.01) and downregulated (combined score < −20) mouse transcriptional regulatory networks from the TRRUST Transcription Factors 2019, according to Enrichr analysis (*38*). (**B**) RT-qPCR in mouse liver of PPAR network genes. (**C**) RT-qPCR of hepatic *Fgf21* mRNA transcripts; *P = 0.0168. (**D**) Circulating serum Fgf21 levels; ****P < 0.0001. Combined score is computed by taking the log of the *P* value from the Fisher exact test and multiplying that by the *z* score of the deviation from the expected rank. Data are presented as mean ± SD. *P < 0.05, **P < 0.01, ***P < 0.001, ****P < 0.0001 (unpaired two-tailed *t* test).

While the effects of many ACC inhibitors have been well characterized (*19*, *20*, *22*), the distinct treatment outcomes of TOFA, especially regarding serum triglycerides and its anti-obesity effects, remain unexplained. To better understand the effects of TOFA on whole genome expression, RNA-seq analysis was performed on liver tissues from DIO mice treated with vehicle or TOFA. Differentially regulated genes (DEGs) were identified, revealing 1,184 DEGs, of which 758 genes were upregulated and 426 were downregulated (fig. S6A). Intriguingly, Enrichr pathway enrichment (*38*) analysis of upregulated genes against the TRRUST 2019 database of transcriptional regulatory networks revealed the enrichment of the PPAR nuclear hormone receptor superfamily as being among the top enriched transcription factors in databases for mouse (Fig. 4A) as well as human (fig. S6B) in response to TOFA treatment. Amongst the PPAR family, *Ppara* mediates the fasting response in liver and plays a key role in regulating genes involved in lipid and energy homeostasis, strongly promoting mitochondrial fatty acid beta-oxidation (*39*–*41*). Notably, the RNA-seq analysis also revealed downregulation of the *Srebp1* transcriptional network, which controls fatty acid and lipid biosynthesis (Fig. 4A), consistent with the lack of hypertriglyceridemia [a metabolic side effect after treatment with other ACC inhibitors as discussed above (*22*)] upon TOFA treatment. RT-qPCR analysis of liver samples showed upregulation of multiple PPAR network genes associated with the hallmark *Ppara*-mediated fasting response (Fig. 4B). Intriguingly, FGF21, a hepatokine regulated by PPARα with relevant pharmaceutical effects in mediating weight loss and pleiotropic metabolic effects of PPARα (*42*), was shown to be significantly upregulated in both mRNA transcripts as well as circulating protein in serum (Fig. 4, C and D). Further RNA-seq analysis of gastrocnemius muscle tissue also revealed strong upregulation of the PPAR signaling pathway by gene set enrichment analysis (GSEA) (*43*) in this metabolic tissue, as well as broad downregulation of inflammatory pathways (fig. S6, C to F). Taken together, these findings suggest that TOFA not only acts through the previously characterized ACC inhibition, but also stimulates the PPAR network, thereby conferring beneficial therapeutic effects promoting an overall favorable anti-obesity and anti-inflammatory profile.

### Activation of PPARα and PPARδ is critical for the full beneficial effects of TOFA treatment

**Fig. 5.**
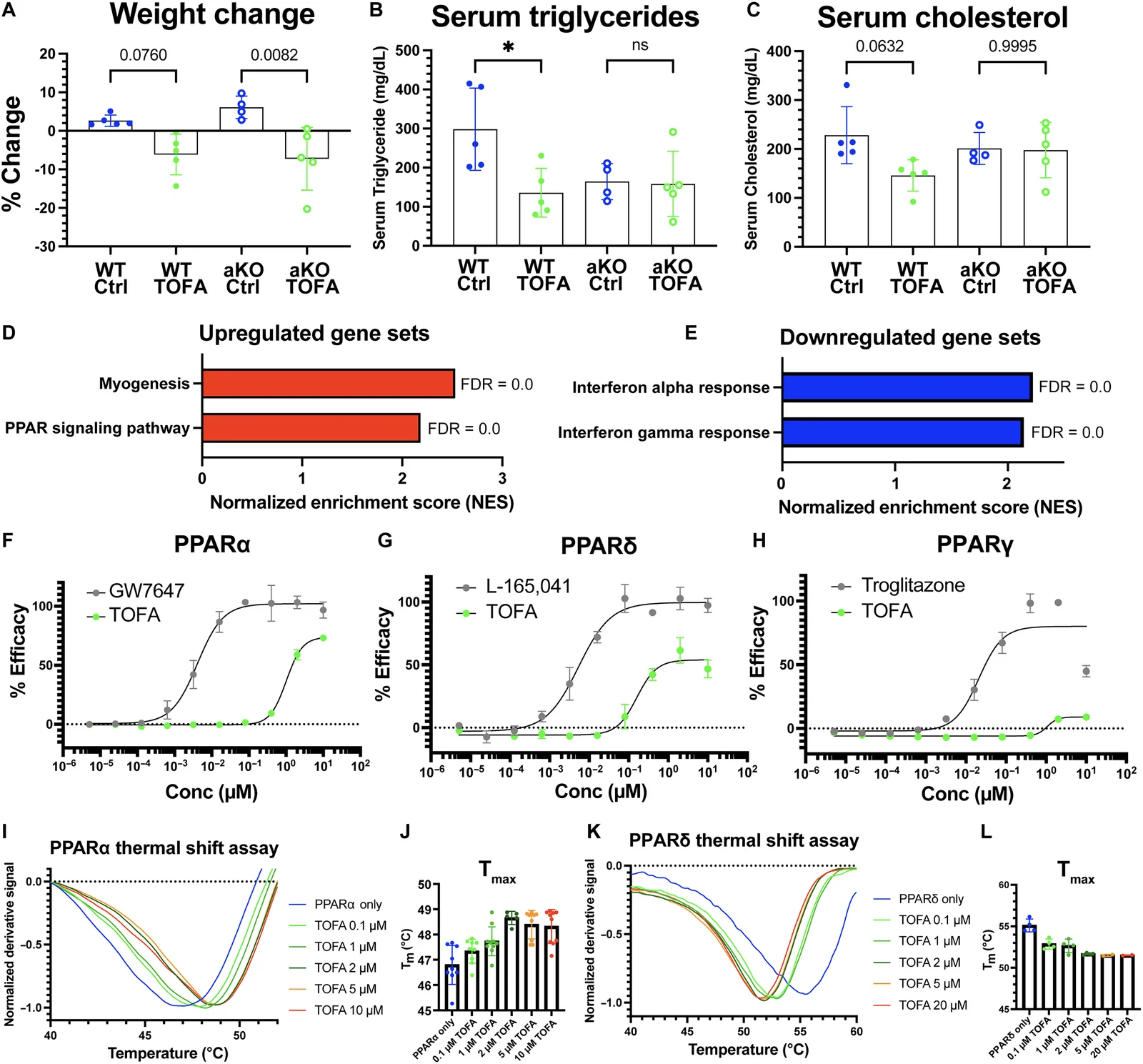
TOFA complementarily bridges ACC inhibition by engaging specific PPAR subtypes. (**A** to **C**) Male C57BL/6 J mice or *Ppara* KO (aKO) mice were placed on a 60% HFD for ten weeks and treated for two weeks with vehicle or TOFA (200 mg/kg/day); *n* = 4–5/group. (A) Body weight change. (B) Serum triglycerides; *P = 0.0251, *P* = 0.9995 (ns). (C) Serum cholesterol. (**D** and **E**) Liver tissue from *Ppara* KO mice were collected, extracted for RNA, and subjected to RNA sequencing and RT-qPCR. (D) Top Hallmark gene sets enriched in genes upregulated by TOFA in *Ppara* KO livers. GSEA (*43*) normalized enrichment score (NES) > 2 and FDR < 0.05. (E) Top Hallmark gene sets enriched in genes downregulated by TOFA in *Ppara* KO livers. GSEA (*43*) normalized enrichment score (NES) > 2 and FDR < 0.05. (**F** to **H**) A cellular assay based on detection of protein-protein interactions between an activated nuclear hormone protein and a steroid receptor co-activator peptide was used to screen various concentrations of TOFA for activity levels against (F) PPARɑ, (G) PPARδ, (H) PPARγ. (**I**) Thermal shift assay displaying PPARɑ protein melting curves in the presence of various concentrations of TOFA and (**J**) temperature at which the maximum (T_max_) dT(fluorescence) signal was observed. *n* = 5–10/group. (**K**) Thermal shift assay displaying PPARδ protein melting curves in the presence of various concentrations of TOFA and (**L**) temperature at which the maximum (T_max_) dT(fluorescence) signal was observed. *n* = 2–4/group. Data are presented as mean ± SD. ns, not significant; *P < 0.05, **P < 0.01 (one-way ANOVA).

PPARs (α, δ, γ) are nuclear receptors for long-chain fatty acids involved in transcriptional control of metabolism, energy balance, and adipose function, with each subtype differing in expression patterns, ligands, and target genes (*39*, *41*, *44*). Given the structural similarity of TOFA to long-chain fatty acids, which are known ligands of PPARs (*41*), we examined the relationship between TOFA and PPAR family members to further characterize the specificity and mechanism underlying TOFA’s treatment outcomes. PPARα is a known therapeutic target (e.g., via fibrates) to modulate lipid homeostasis in mice and humans by decreasing serum triglyceride levels and increasing fatty acid clearance by beta-oxidation, primarily through effects in the liver (*45*). To evaluate the possible role of PPARα in mediating metabolic effects of TOFA, whole body *Ppara* knockout (KO) mice were fed a 60% HFD for ten weeks followed by a two-week oral treatment of vehicle or TOFA daily. TOFA reduced body weight in both wild-type and *Ppara* KO mice (Fig. 5A). However, in the wild-type mouse, TOFA treatment decreased serum triglycerides but this decrease was blunted in TOFA-treated *Ppara* KO mice compared to control treatment (Fig. 5B). Similarly, serum cholesterol levels presented the same pattern in which the *Ppara* KO condition diminished the TOFA-dependent decrease in serum cholesterol (Fig. 5C). RNA-seq analysis of liver tissue from *Ppara* KO mice revealed the PPAR signaling pathway as a persistently upregulated pathway in gene set enrichment analysis (Fig. 5D), as well as interferon-related responses as top downregulated gene sets (Fig. 5E). Amongst other gene sets, we observed a consistent increase in fatty acid metabolism hallmark gene signature (fig. S7A) and a decrease in inflammatory response hallmark gene signatures (fig. S7B). The upregulated PPAR signaling pathway (fig. S7C) in *Ppara* KO mice treated with TOFA pointed to a redundant or compensatory PPAR family member at play in the liver, such as *Ppard* (fig. S7D). Subsequent analysis by RT-qPCR revealed genes, such as *Angptl4*, *Fabp4*, and *Cyp4a14*, that share multiple PPAR subtype transcriptional regulatory elements were persistently upregulated by TOFA treatment in *Ppara* KO mice (fig. S7E). However, several PPAR target genes, such as *Ucp2*, *Pdk4*, and *Cd36*, previously upregulated by TOFA treatment in wild-type DIO mice were no longer upregulated in *Ppara* KO mice (fig. S7F). The attenuated yet persistent upregulation of specific PPAR target genes in *Ppara* KO mice suggests functional redundancy among PPAR subtypes in the liver, primarily PPARα and PPARδ due to their prominent hepatic expression (*39*, *46*). Furthermore, analysis of gastrocnemius muscle, with strong PPARδ expression (*47*), showed upregulation of PPARδ target genes, such as *Pdk4* and *Cpt1b*, by TOFA treatment (fig. S7, G and H), suggesting a role for PPARδ agonist activity inducing peripheral energy metabolism and having a positive effect on mitochondrial respiratory function (*48*–*51*). To confirm this hypothesis, we utilized a cell-based protein-protein interaction assay to assess receptor activation and subsequent co-activator recruitment. TOFA showed strong agonist activity against human PPARα (EC_50_ = 2.095 μM) and human PPARδ (EC_50_ = 0.594 μM), with very weak to no activity on human PPARγ (EC_50_ > 10 μM) (Fig. 5, F to H). Furthermore, activation of PPARα and PPARδ was shown to maximize at 74% and 54%, respectively, relative to full agonist controls, implying partial PPARα/δ agonist activity of TOFA (Fig. 5, F to H). To further investigate the mechanistic relationship between TOFA and PPARα and PPARδ, biochemical thermal shift assays with purified ligand-binding domains revealed that TOFA directly interacted with both PPARα and PPARδ proteins in a concentration-dependent manner. TOFA binding stabilized the PPARα LBD, producing a significant increase in melting temperature from 1 μM onward (Fig. 5, I and J) while TOFA induced a significant decrease in the melting temperature of the PPARδ LBD from 0.1 μM onward (Fig. 5, K and L), indicative of a ligand-induced conformational rearrangement. Taken together, these findings suggest that the persistent hepatic PPAR transcriptional signal in TOFA-treated *Pparɑ* KO mice results from residual Pparδ activity, highlighting the importance of the PPAR network, specifically TOFA interaction with PPARα and PPARδ, in mediating the improvements in lipid and energy metabolism from TOFA treatment.

### Synergistic effects of ACC1/2 inhibition and PPARα/δ agonism in treatment outcomes of TOFA

Given the dual action of TOFA, we aimed to determine the proportional contributions of ACC inhibition versus PPAR agonism on metabolic improvements. To test whether TOFA achieves greater outcomes compared to single agent treatment with mechanistic mimics, mice fed with a HFD were treated with either vehicle control, TOFA, Firsocostat (clinical ACC1/2 inhibitor), Elafibranor (clinically approved dual PPARα and PPARδ agonist), or a combination of Firsocostat and Elafibranor. Consistently, TOFA treatment outperformed both single and combination regimens, especially compared to Firsocostat, which elevated body weight and serum triglyceride levels. The negative effects of Firsocostat treatment were corrected by co-treatment with the PPARα/δ agonist Elafibranor, likely through Pparα activation (fig. S8, A to C). Notably, even as a single agent, TOFA treatment led to greater body weight reduction than Elafibranor alone or the combination of Elafibranor with Firsocostat (fig. S8, A and B). The hypertriglyceridemia in Firsocostat-treated mice was correlated with an increase in *Srebp1c* mRNA expression, which was not observed with other treatment regimens (fig. S8D). Furthermore, Firsocostat treatment did not impact fasting blood glucose or insulin levels while TOFA, Elafibranor, and combination treatment (FIR/ELA) reduced both, contributing to decreased risk of insulin resistance and diabetes progression, and suggesting the mechanistic dependence of PPARα and PPARδ signaling (fig. S8, E and F). With regards to liver triglyceride levels, TOFA and Firsocostat were the most effective single agents in reducing hepatic triglycerides, suggesting mechanistic ACC inhibition dependence in decreasing hepatic steatosis. In contrast, the PPARα/δ agonist Elafibranor showed only marginal effects on hepatic steatosis when used alone (fig. S8G). Accordingly, combination of Elafibranor with Firsocostat produced only a small decrease in liver triglyceride levels compared to Firsocostat alone (fig. S8G). Moreover, treatment of human Huh7 hepatoma cells with Firsocostat failed to elevate canonical PPARα/δ target genes *CPT1A and VLDLR* while both PPARα/δ-acting Elafibranor and TOFA do (fig. S8, H and I), demonstrating ACC-independent PPARα/δ target activation and consistent with our previous observation of PPARα/δ-dependence for energy expenditure-related transcriptional responses. Therefore, TOFA bridges the individual, beneficial actions of ACC inhibition and PPARα/δ activation and overcomes target-class shortcomings in one molecule by a synergistic dual-target mechanism.

### TOFA treatment blunts MASLD/MASH development

**Fig. 6.**
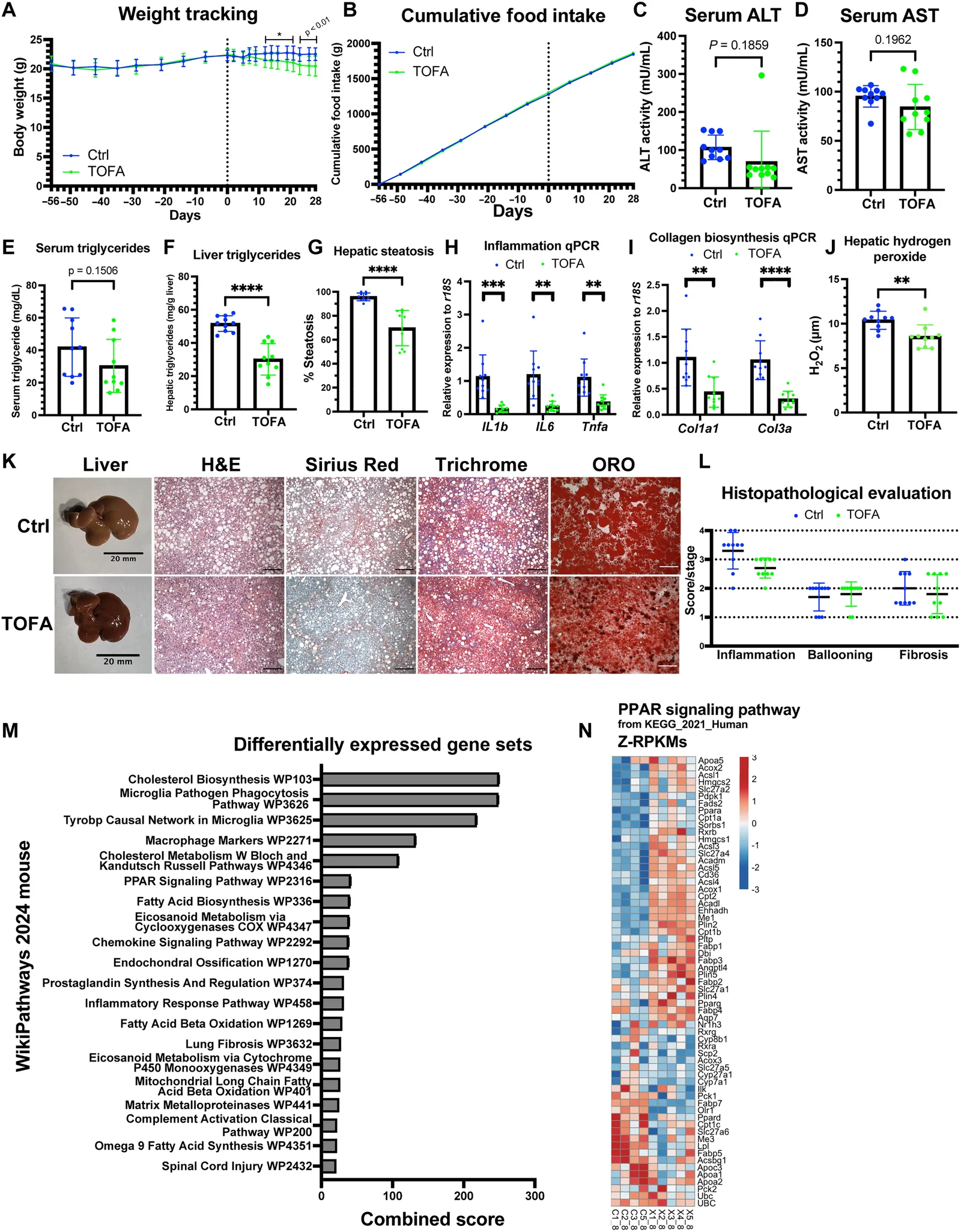
Oral TOFA treatment resolves MASLD/MASH-related inflammation, hepatosteatosis, and fibrosis. [(A) to (N)] Male C57BL/6 J mice were placed on a CDAA-HFD for eight weeks and treated for four weeks with vehicle or TOFA (125 mg/kg, BID); *n* = 10/group. (**A**) Body Weight; *P < 0.05. (**B**) Food Intake. (**C**) Serum ALT. (**D**) Serum AST. (**E**) Serum triglycerides. (**F**) Liver triglycerides; ****P = 4.975E-06. (**G**) Hepatic steatosis; ****P = 3.46E-05. (**H**) Quantitative real-time PCR of the indicated hepatic mRNAs for inflammation; ***P = 0.00025, **P = 0.001, **P = 0.0011. (**I**) Quantitative real-time PCR of the indicated hepatic mRNAs for collagen biosynthesis; **P = 0.003, ****P = 0.00002. (**J**) Hepatic hydrogen peroxide; **P = 0.0026. (**K**) Representative whole liver, H&E, Sirius Red, Trichrome, and Oil Red O (ORO) staining of liver sections. Scale bars, 20 mm or 200 μm (inset). (**L**) MASH activity scores. (**M** and **N**) Liver tissue from mice were collected, extracted for RNA, and subjected to RNA sequencing. (M) Top 20 transcriptional regulatory networks enriched in DEGs, according to Enrichr analysis (*38*) against the WikiPathways database, with a combined score > 20 and adjusted *P* value <0.01. (N) Heat map of genes defined in the PPAR signaling pathway from KEGG_2021_Human, with a *P* < 0.0001 for the upregulated response. Columns represent samples and rows represent genes. Combined score is computed by taking the log of the *P* value from the Fisher exact test and multiplying that by the *z* score of the deviation from the expected rank. Data are presented as mean ± SD. *P < 0.05, **P < 0.01, ***P < 0.001, ****P < 0.0001 (unpaired two-tailed *t* test).

Beyond its metabolic benefits in diet-induced obesity, glucose intolerance and insulin resistance, we evaluated TOFA in mouse models of MASLD/MASH. Male C57BL/6 J mice were fed a choline-deficient, L-amino acid-defined, high-fat diet (CDAA-HFD, a classic MASLD/MASH mouse model (*52*)) for eight weeks followed by treatment with vehicle or TOFA for four weeks. TOFA-treated mice demonstrated weight loss, with a significant change by day 14 that was sustained until end of treatment with an average weight loss of 8.7% (Fig. 6A). Overall, TOFA was orally tolerated as evidenced by no change in food intake (Fig. 6B). TOFA treatment also resulted in a trending decrease in serum ALT and AST, circulating markers of liver damage, compared to vehicle control (Fig. 6, C and D and fig. S9, A and B). Serum triglyceride levels were also reduced (Fig. 6E), however the lack of lipoprotein production due to dietary choline deficiency blunts the severity of hypertriglyceridemia in this model (*53*). Similar to our observations in DIO mice, TOFA significantly reduced liver triglyceride levels in this MASLD/MASH model (Fig. 6F and fig. S9C). The reduction of total hepatic lipid content directly correlated with ameliorated hepatic steatosis, revealed by histological staining (Fig. 6G). The expression of various inflammatory genes such as *Il1b*, *Il6*, and *Tnfa*, hallmarks of MASH, were also markedly and significantly reduced in the liver, consistent with a potent anti-inflammatory effect of TOFA treatment (Fig. 6H). Additionally, expression of collagen biosynthesis genes *Col1a1* and *Col3a* was significantly reduced, indicating a key anti-fibrotic benefit of TOFA, targeting another hallmark of MASH (Fig. 6I). Complementary to the improvements in hepatic inflammation and fibrotic markers, livers of TOFA-treated mice had significantly lower hepatic hydrogen peroxide content, indicating decreased oxidative stress and damage (Fig. 6J). Overall, there was marked improvement in liver histology and decreased lipid accumulation (by H&E and Oil Red O) and fibrotic marker staining (by Sirius Red and Masson Trichrome) (Fig. 6K). Further histopathological scoring of livers was performed using a standardized scoring system for MASLD/MASH activity (*54*). Of the MASH components, overall decreases in inflammation and fibrosis were observed, with a modest effect on ballooning (Fig. 6L). The improvements in hepatic steatosis, inflammation, and collagen deposition in response to TOFA treatment were also found to be dose-dependent (fig. S9, D to G).

RNA-seq analysis of liver tissues from CDAA-HFD mice and subsequent Enrichr analysis using the WikiPathways 2024 Mouse database identified several lipid metabolism pathways, including PPAR signaling and fatty acid beta-oxidation, as enriched among these DEGs, consistent with the PPAR transcriptional signature observed in the DIO model (Fig. 6M). Accordingly, many of the top upregulated genes were associated with the PPAR signaling pathway, as identified by Enrichr analysis using KEGG 2021 Human dataset (Fig. 6N). Given the ongoing clinical trials of ACC inhibitors for MASLD/MASH, TOFA efficacy was benchmarked against Firsocostat efficacy in the CDAA-HFD model. TOFA-treated mice showed a more pronounced reduction in body weight (fig. S10A) without any changes in food intake (fig. S10B) and serum triglycerides (fig. S10C). TOFA treatment demonstrated similar efficacy as Firsocostat on MASLD/MASH disease markers, including serum ALT and AST (fig. S10, D and E), liver triglycerides (fig. S10F), and biomarkers of inflammation and collagen biosynthesis (fig. S10, G to J).

The *MUP-uPA* mouse fed a HFD is a well-established transgenic model for studying MASLD/MASH, as it induces chronic liver injury by triggering ER stress specifically in hepatocytes through the expression of urokinase-type plasminogen activator (uPA) under the liver-specific *Mup* promoter (*55*). The *MUP-uPA* model overcomes limitations of diet-induced models by combining metabolic features with advanced MASH progression, including bridging fibrosis, to better reflect human MASH pathological manifestations. After ten weeks of HFD feeding to accelerate disease progression, male *MUP-uPA* mice were treated with either vehicle control or TOFA for an additional six weeks. During the treatment period, TOFA-treated mice experienced significant weight loss by two weeks and sustained through the remainder of the treatment time course, with no changes in food intake (fig. S11, A and B). Consistent with our previous results in other diet-induced disease models, TOFA-treated mice exhibited an overall decrease in triglyceride levels, in both liver and serum, as well as a decrease in hepatic cholesterol (fig. S11, C to F). Liver histology revealed profound decreases in lipid accumulation by Oil Red O staining as well as a marked decrease in fibrosis as assessed by Sirius Red staining (fig. S11, G to I). RNA-seq analysis of liver samples further highlighted the strong upregulation of genes related to lipid metabolism and marked downregulation of a wide array of inflammatory signaling pathways (fig. S11, J and K). Taken together, TOFA was effective in addressing hepatic steatosis, inflammation, and fibrosis in this severe MASLD/MASH model.

### TOFA enhances anti-obesity profile quality and persistence as a complementary agent to incretin analogs

**Fig. 7.**
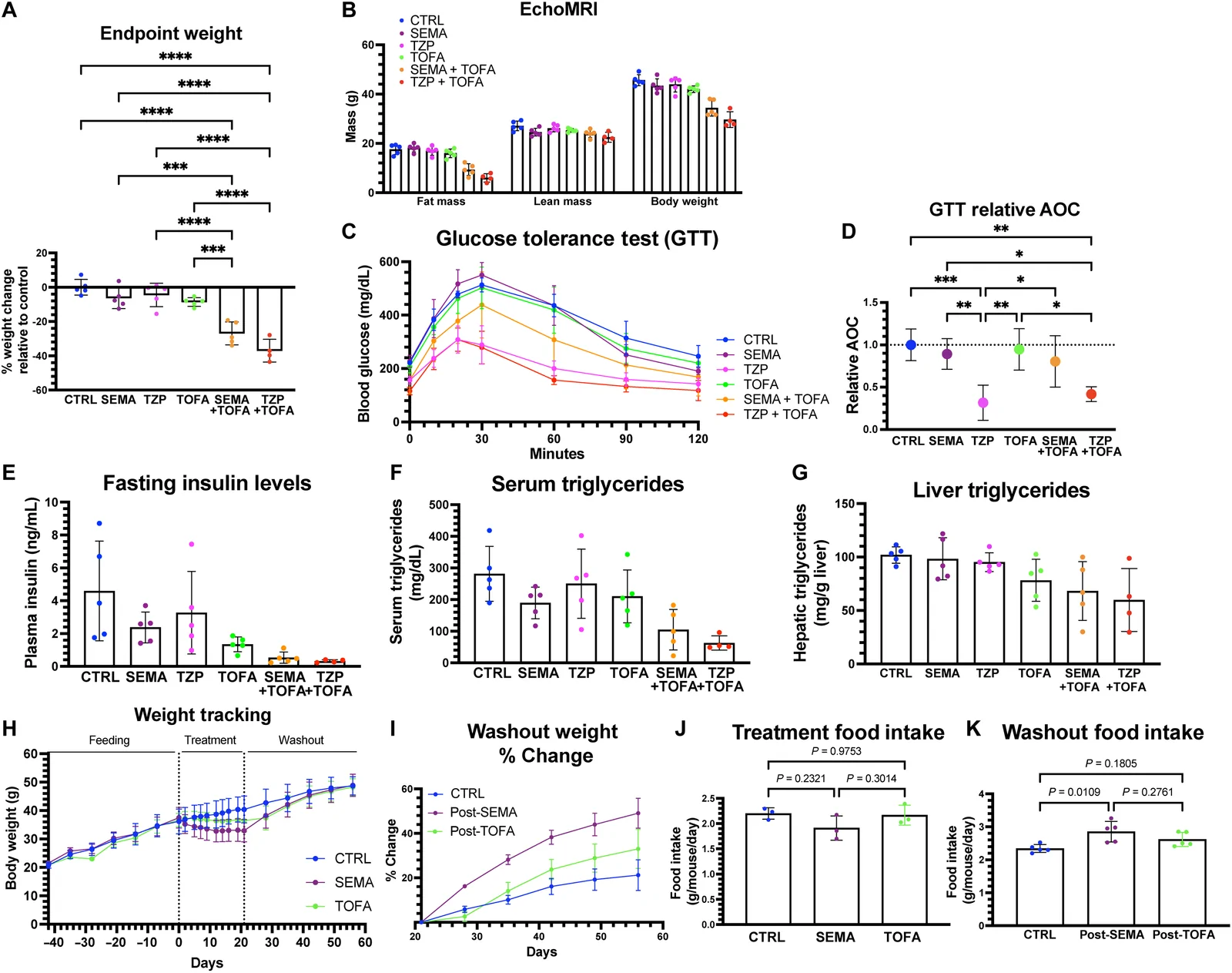
TOFA action is complementary to incretin analogs. [(**A**) to (**G**)] Male C57BL/6 J mice were placed on a 60% HFD for twelve weeks and treated for 24 days with vehicle, oral TOFA gavage (200 mg/kg/day), semaglutide injection (0.5 nmol/kg/day), tirzepatide injection (0.5 nmol/kg/day), combination of TOFA gavage and semaglutide injection, or combination of TOFA gavage and tirzepatide injection; *n* = 4–5/group. To avoid severe, rapid onset weight loss, tirzepatide and TOFA combination treated mice were shifted to maintenance dosing of half concentrations by Day 10. (A) Total treatment body weight change, relative to control group. (B) EchoMRI body composition analysis. (C) Glucose tolerance test. (D) Relative area of the curve (AOC) to control from glucose tolerance test (GTT). (E) Fasting blood insulin levels. (F) Serum triglycerides. (G) Liver triglycerides. (**H** to **K**) Male C57BL/6 J mice were placed on a 60% HFD for six weeks and treated for three weeks with vehicle, oral TOFA gavage (200 mg/kg/day), or semaglutide injection (10 nmol/kg/day). At the end of treatment, mice were monitored for an additional five weeks; *n* = 5/group. (H) Body weight. (I) Body weight change post-treatment. (J) Food intake during treatment. (K) Food intake posttreatment. Data are presented as mean ± SD. *P < 0.05, **P < 0.01, ***P < 0.001, ****P < 0.0001 (one-way ANOVA).

While targeting energy intake to improve metabolic health has seen clinical success with incretin-based therapies (*56*, *57*), targeting energy expenditure is an alternative, promising path for obesity treatment that could be complementary in combination with existing incretin-based approaches. We explored the potential for combinatorial effects of TOFA with incretin analogs in DIO mice with daily treatment of vehicle control, subcutaneous injection of minimum efficacious doses of semaglutide (Novo Nordisk) or tirzepatide (Eli Lilly), oral gavage of a sub-optimal dose of TOFA, or combinations for 24 days. Upon treatment initiation, tirzepatide and TOFA combination-treated mice experienced rapid onset weight loss requiring moving to lower maintenance dosing. Semaglutide and TOFA-treated mice demonstrated a slower, steady weight loss after a lag time of about a week. Overall, all monotherapy treatments demonstrated similar degrees of weight modulation relative to the controls (average 10% weight loss compared to controls), while the combination treatments significantly outperformed the monotherapies in weight loss (Fig. 7A). The significant changes in body weight were solely due to fat mass loss, with no significant loss of lean mass (Fig. 7B). Tirzepatide treatment alone strongly promoted improved glucose handling, and combination treatments (TOFA + TZP and TOFA + SEMA) demonstrated stronger improvements in glucose control (Fig. 7, C and D). Fasting insulin levels were also most improved in the combination treatment groups (Fig. 7E). Similarly, serum and hepatic triglycerides presented the greatest decreases in combination treatments, outperforming the single agent treatment effects (Fig. 7, F and G). Given the distinct mechanism of action between TOFA and incretin analogs, we investigated durability of weight loss by assessing time to weight rebound after treatment termination of semaglutide or TOFA fed a 60% HFD (Fig. 7H). After treatment termination, or the washout period, TOFA-treated animals were able to maintain body weight levels similar to that of control-treated mice while semaglutide-treated subjects experienced an immediate and stark body weight increase (Fig. 7I). As expected, body weight loss during semaglutide treatment was attributed to decreases in food intake, with body weight regain after treatment termination associated with significant increases in food intake; by contrast, no changes in food intake were observed between TOFA-treated and vehicle-treated mice (Fig. 7, J and K). Taken together, TOFA represents a complementary agent in combination with incretin analogs to promote greater weight loss and improve sustainability of metabolic and lipid health restoration independent of appetite-regulating mechanisms.

We also explored the use of semaglutide alone and in combination with TOFA in MASLD/MASH using the CDAA-HFD model. Consistent with previous results, the combination treatment yielded a greater degree of weight loss (fig. S12, A and B), greater decrease in lipid levels in the liver (fig. S12C), improvements in liver histopathology (fig. S12, D to G), and strong reductions in inflammation (fig. S12, H and I) and fibrosis (fig. S12, J and K) markers. These additional studies further highlight the complementary metabolic actions of TOFA to the current class of satiety-inducing incretin analogs that could be used in combination to outperform shortcomings of single agent treatments.

## DISCUSSION

Overweight, obesity, and associated metabolic dysfunction are growing in prevalence worldwide and represent important public health threats. The heterogeneity of metabolic disease manifestations, such as obesity, insulin resistance, dyslipidemia, and hepatic steatosis, further complicates finding holistic treatments. The recent regulatory approvals of semaglutide (Novo Nordisk), an appetite suppressing GLP-1 incretin analog, for multiple metabolic indications, such as type 2 diabetes mellitus, weight management, and most recently, MASH, is a promising development but it is also associated with undesirable side effects, such as loss of muscle mass. Multi-targeting compounds, as seen with the FDA-approved incretin analog tirzepatide (Eli Lilly) that targets receptors for both GLP-1 and GIP, and retatrutide (in human phase 3 trials, Eli Lilly), which targets GLP-1, GIP, and GCG receptors, reflect growing acceptance of the need to address complex metabolic disease with therapeutics acting through multiple mechanisms. While the approvals and late clinical development of incretin analogs remain a notable area of interest, these incretin-targeting products remain limited to mechanisms related to satiety and hormonal appetite regulation. Identification and development of biological targeting strategies would benefit and complement the current landscape of metabolic disease management drugs.

In this study, we characterize TOFA as an orally bioavailable ACC1/2 inhibitor with partial PPARα/δ agonism, a synergistic combination of clinically validated targets conferring unexpectedly potent benefits in countering obesity and its comorbidities, alone or with co-administered incretin analogs. Previous studies have shown that ACC1/2 inhibition alone fails to reduce weight or improve insulin resistance, and is associated with significant elevation of circulating triglycerides (*21*, *58*). This raised the question regarding TOFA’s differentiated mechanism of action. Utilizing RNA-seq analysis combined with in vivo whole-body *Ppar*α knockout, we revealed a unique role of TOFA acting to stimulate both the PPARα and PPARδ nuclear receptors, further confirmed by data from receptor-specific interaction studies. Therapeutic activation of PPAR subtypes has demonstrated clinical efficacy to treat metabolic diseases including dyslipidemia, insulin resistance, and disordered lipid homeostasis (*44*). This includes fibrates (PPARα agonists) and thiazolidinediones (TZDs, PPARγ agonists), as well as PPARδ-activating compounds such as Elafibranor (PPARα/δ dual agonist) and Seladelpar (PPARδ agonist), approved for the treatment of primary biliary cholangitis (*59*–*62*). While a previous study suggested a potential indirect link between TOFA and PPARα activation (*63*), we document here a direct biochemical interaction of TOFA with the ligand-binding domains of both PPARα and PPARδ, and describe the functional involvements of these transcription factors in mediating TOFA’s metabolic effects both in vitro and in vivo. We speculate that TOFA’s differentiated treatment outcomes as compared with other ACC inhibitors arise from the synergistic and nonredundant activities of full ACC1/2 inhibition (countering lipid production and promoting fatty acid beta-oxidation) and partial agonist activity against PPARα and PPARδ (mediating weight loss and improved type 2 diabetes-related phenotypes). In vivo co-administration of Firsocostat (ACC1/2 inhibitor) and Elafibranor (PPARα/δ dual agonist) failed to phenocopy the metabolic improvements seen with TOFA, indicating that engaging both target classes with separate agents is insufficient. Complementary in vitro experiments showed that TOFA induces PPAR target genes independently of ACC inhibition, identifying the PPAR arm as a direct and required contributor to the energy expenditure response rather than a downstream consequence of ACC blockade. Together, our studies resolve the conundrum of why TOFA does not elevate blood lipids (a known ACC1/2 inhibitor class effect) and is able to beneficially impact energy expenditure.

Indeed, known downstream targets of PPARα, such as FGF21 (*42*), may help to further clarify the unexpected energy expenditure effects of TOFA by providing an avenue for stimulating mitochondrial energy output. FGF21 hormone analogs have been shown to promote improvements on liver metabolic health in diet-induced obese mice and in human clinical trials (*64*, *65*). Consistent with the observed increased circulating FGF21 in response to TOFA treatment in mice, FGF21 may indeed promote browning activation of brown and beige thermogenic tissues (*66*). Although the modest increase in *Ucp1* in BAT/WAT in response to TOFA treatment is suggestive of an uncoupling thermogenic program in adipose tissue, our metabolic cage data show elevated energy expenditure more prominently under room temperature and thermoneutral conditions as compared with cold exposure, a classic thermogenic stressor. Hence, Ucp1-linked thermogenesis is unlikely to fully account for the broad stimulation of energy expenditure. Increased expression of *Ucp2* and *Ucp3* in liver and muscle of TOFA-treated mice and elevated mitochondrial activity in hepatocytes in response to TOFA treatment might support additional mechanisms promoting whole-body energy expenditure. While the physiological functions of Ucp2 and Ucp3 remain less clear as compared to Ucp1, the upregulation of Ucp2 and Ucp3 may contribute to decreases in the ATP/ADP ratio, stimulate the starvation response, modulate reactive oxygen species production, and protect against insulin resistance (*67*, *68*).

Previous development of pan-PPAR agonist compounds has shown modest or neutral effects on body weight, speculated to be due to lower dosing regimens required to avoid toxicity resulting from full agonist activity (*69*). The partial agonistic characteristic of TOFA may allow for a higher therapeutic dosing window for optimal, strong PPAR signature activities, such as promotion of weight loss, without incurring undesirable side effects associated with full activation of the PPAR network (*70*, *71*). Furthermore, the apparent lack of activity on PPARγ by TOFA is ideal given known class side effects (such as edema and weight gain) associated with PPARγ activation (*72*). It is still unclear whether the partial activity of each respective PPAR subtype may be compensatory or complementary to each other, especially given the known synergistic roles PPARα and PPARδ play together (*69*). PPARs bind to and are activated by fatty acids, and consistent with the unique structural carboxylic acid carbon tail on TOFA resembling a fatty acid (*73*–*76*), we demonstrate that TOFA acts as a direct ligand for PPARα and PPARδ as verified by biochemical thermal shift assays with purified proteins. This activity is consistent with recent reports that other fatty acid-like small molecules, including bempedoic acid and the enedioic acid analog 326E, also engage and activate PPARα (*77*, *78*). TOFA is also converted intracellularly to TOFyl-CoA, an allosteric inhibitor that competes with acetyl-CoA in the ACC1/2 carboxyltransferase reaction (*32*, *33*, *79*–*81*). We have verified that TOFyl-CoA exhibits greater potency in vitro as an ACC1/2 inhibitor as compared with TOFA, however, the relevance of these findings to in vivo physiology remains to be determined. Our findings demonstrate that TOFA engages both ACC1/2 inhibition and PPARα/δ agonism within a single molecule that produces synergistic metabolic benefits, including increased energy expenditure and improved blood lipid profiles, that are not achievable through either mechanism targeted alone.

Given TOFA’s energetic and metabolic effects, we hypothesized that appetite-regulating incretin analogs, which reduce energy intake, could synergize with TOFA’s energy expenditure boost when co-administered. This type of multi-targeting combinatorial approach has recently been explored in early preclinical and clinical studies, especially in disease areas beyond diabetes and weight management, such as fatty liver diseases (*82*–*84*). While our data confirm that both incretin analogs and TOFA independently beneficially impact metabolic health, our studies show combination treatment of incretin analogs and TOFA may present an opportunity to overcome shortcomings of incretin analog treatments such as loss of muscle mass, especially relevant in older patient populations facing sarcopenia (*85*, *86*). Another concern regarding incretin analog treatments is weight rebound after treatment termination (*87*). Our studies show that TOFA-treated mice were able to sustain their end-of-treatment body weight for a longer period of time compared to semaglutide-treated mice. Additionally, our data showing that TOFA is effective as an orally bioavailable agent may overcome the efficacy gap of the oral forms of incretin analogs, which have limited bioavailability and efficacy, requiring oral enhancers and dosing at far higher levels (*88*). Importantly, due to their synergistic effects, combination treatments of TOFA with incretin-based therapeutics could allow lower and less frequent dosing, and potentially decrease side effects associated with the incretin analog mechanism of action, such as muscle mass loss, gastrointestinal side effects and post-treatment weight rebound.

While TOFA in our current studies showed impressive therapeutic efficacy across multiple severe metabolic conditions, there are still additional considerations for clinical translation. We cannot rule out indirect involvement of other metabolic targets or additional TOFA-action in other metabolically active cells and tissues with PPARα/δ and ACC1/2 expression, such as adipocytes or skeletal muscle, with anti-obesity effects. Male mice were employed for all in vivo studies due to protective effects of estrogen in female mice against obesity-associated hepatic and muscle insulin resistance by suppressing white adipose tissue lipolysis and subsequent lipid deposition in muscle and liver tissue (*89*, *90*). Follow-up studies in female animals should evaluate anti-obesity and metabolic disease modulation efficacy as well as safety of TOFA. While the results from the dose-dependence studies of TOFA in DIO (fig. S2) and MASLD/MASH (fig. S9) models suggest that lower titration of doses may confer more gradual, gentler outcomes especially in regard to body weight loss rate, a minimal efficacious dose for weight loss is still yet to be determined. In our various efficacy studies, doses in the range of 62.5–200 mg/kg were primarily employed either once daily or twice daily regimens, which equates to an approximate range of 5–16 mg/kg for human dosing by allometric scaling (roughly equivalent to 500–1600 mg per 100 kg human patient) (*91*). By reference, Vascepa (icosapent ethyl) for triglyceride management is dosed at 4 grams per day, showing potential feasibility for TOFA dosing amounts, pending safety studies. In the present studies, the safety profile at doses used is incompletely characterized for subclinical toxicity. Encouragingly, previously published data on TOFA demonstrate excellent tolerability, with an undefined LD50 (oral LD50 > 5 g/kg in rats), and six-months chronic dosing in rats showing no obvious signs of toxicity (*27*). The strong safety profile suggests the feasibility of finding a therapeutic efficacy dose window for TOFA, allowing its dual target engagement, and suggesting that anti-obesity efficacy of TOFA may not be limited to the tested doses in our present studies. We expect additional data from ongoing studies on exposure levels as well as pharmacokinetic or pharmacodynamic data in higher species to better define dosing concentration and frequency for TOFA for clinical translation. Moreover, functional studies and safety evaluation in nonhuman primates and humans will need to verify the translatability of our findings to higher species. Given these limitations, we acknowledge that molecular mechanisms linking TOFA to restoring metabolic homeostasis may require further investigation to draw additional conclusions for clinical uses.

Our data in aggregate support consideration of TOFA for further preclinical development and clinical evaluation to address the growing and unmet medical need of patients with obesity and related metabolic disorders. Pending animal and human safety and efficacy trials, therapeutic use of TOFA may be poised to decrease the need for treatment with multiple therapeutic agents to address the multidimensional facets of metabolic disease as well as potentially increase patient compliance as an orally bioavailable option with fewer side effects. Overall, TOFA is a unimolecular multi-specific agent synergistically targeting multiple pathways in energy and lipid metabolism and represents a promising candidate and strategy for weight loss and metabolic disease management.

## MATERIALS AND METHODS

All key reagents and resources used in this study are listed in Table 1.

**Table 1.** Key reagents and resources used in this study, including supplier details and relevant identifiers.

REAGENT or RESOURCES	SOURCE	IDENTIFIER
Chemicals, Peptides, and Recombinant Proteins		
Poly(ethylene glycol); PEG200	Sigma	P3015
1-methyl-2-pyrrolidinone (NMP)	Sigma	328634
TOFA	Cayman Chemicals	10005263
TOFyl-CoA	DSK Innosciences	DSK-3516
Firsocostat	MedChemExpress	HY-16901
Elafibranor	MedChemExpress	HY-16737
Semaglutide	Novo Nordisk Resource Sharing	
Tirzepatide	MedChemExpress	HY-P1731
Dextrose	Fisher Scientific	D16–500
Humulin R U-100	Lilly	HI-210
QIAzol	Qiagen	79306
PowerUp SYBR Master Mix	Applied Biosystems	A25742
PF-05175157	MedChemExpress	HY-12942
CP-640186	Cayman Chemicals	17691
Human PPARα protein ligand binding domain (amino acids 200–468 with N-terminal 6xHis-tag)	Papa *et al.* (*77*)	N/A
Human PPARδ, Ligand Binding Domain, GST Tag Recombinant Protein	Thermo Fisher Scientific	PV4694
		
Critical Commercial Assays		
RNeasy Kit	Qiagen	74106
iScript Reverse Transcription Supermix for RT-qPCR	BioRad	1708841
Seahorse Mitochondrial Stress Test	Agilent	103015–100
Pierce BCA Protein Assay Kit	Thermo Scientific	23225
Triglyceride Assay Kit	Abcam	ab65336
Cholesterol/Cholesteryl Ester Assay Kit	Abcam	ab65359
Cholesterol Assay Kit – HDL and LDL/VLDL	Abcam	ab65390
ALT Activity Assay	Abcam	ab105134
AST Activity Assay	Abcam	ab105135
Urea Assay Kit	Abcam	ab83362
Creatinine colorimetric assay	Cayman Chemicals	700460
Mouse FGF-21 ELISA	Abcam	ab212160
Mouse Insulin ELISA	CrystalChem	90080
KAPA RNA HyperPrep Kit with RiboErase (HMR)	Roche	KK8561
Acetyl-CoA Carboxylase 1 (ACC1) Assay Kit	BPS Bioscience	79315
Acetyl-CoA Carboxylase 2 (ACC2) Assay Kit	BPS Bioscience	79282
ADP-Glo Kinase Assay	Promega	V6930
Hydrogen Peroxide Assay Kit	Abcam	102500
Protein Thermal Shift Dye Kit	Applied Biosystems	4461146
		
Deposited Data		
RNA-seq dataset	GEO	GSE302447
		
Experimental Models: Cell Lines and Primary cells		
HepG2	ATCC	HB-8065
Huh7	UC Berkeley Cell Culture Facility	CVCL_0336
Primary murine hepatocytes	This study	N/A
		
Software and algorithms		
Prism 10	GraphPad	N/A

### Experimental model and subject details

#### Cell lines

HepG2 or Huh7 cells were obtained from ATCC or UC Berkeley’s Cell Culture Facility, respectively, and propagated according to manufacturers’ instructions.

#### Primary cells

Primary mouse hepatocytes were obtained from adult mouse subjects by liver perfusion and maintained in DMEM supplemented with 10% fetal bovine serum (FBS).

#### Mice

The C57BL/6 J (strain 000664), DIO (strain 380050), and *Ppara* KO (strain 008154) mice were purchased from the Jackson Laboratory, Bar Harbor, ME. C57BL/6 J mice were aged 6–8 weeks and DIO mice were aged 16–18 weeks. *Ppara* KO breeder pairs were maintained at UC Berkeley’s animal facilities. All experimental procedures were conducted in accordance with IACUC regulation and were approved by UC Berkeley’s IACUC (Protocol # AUP-2018-10-11513-2).

### Method details

#### Mouse diet-induced obesity studies

Experiments were performed using 6-week-old male C57BL/6 J mice purchased from The Jackson Laboratory, Bar Harbor, ME. The diet-induced obese (DIO) mice were fed a diet containing 60% (kcal) fat (D12492i, Research Diets) for 7–12 weeks before and thereafter during treatment. After reaching study design start for treatment, mice were treated with either vehicle control (5% NMP, 20% PEG-200) or TOFA either once (OD) or twice (BID) daily, as described in study designs. Body weight was measured every week. During the treatment, glucose and insulin tolerance tests were formed after three weeks of treatment and body composition was measured after four weeks of treatment. Metabolic activity of mice was analyzed in metabolic cages after four weeks of treatment and data was collected for four days. Upon sacrifice, liver, brown and white adipose depots (inguinal and epididymal), and muscle (quadriceps, gastrocnemius) were collected and ∼1 mL of blood was obtained from each mouse by cardiac puncture. Blood was centrifuged at 2,000 g for 5 min at 4°C to obtain serum, which was frozen at −80°C. All mouse procedures were approved by the University of California, Berkeley Institutional Animal Care and Use Committee (Protocol # AUP-2018-10-11513-2). All protocols conform to federal regulations, the National Research Council Guide for the Care and Use of Laboratory Animals, and the Public Health Service Policy on Humane Care and Use of Laboratory Animals.

For benchmarking and combination studies, dosing of known clinical compounds in vivo were informed by existing literature references, where Firsocostat and Elafibranor were dosed at an optimal or similar efficacious dose (*23*, *62*, *92*, *93*). Semaglutide and tirzepatide were both dosed daily [due to the relatively short half-life of incretin analogs in mice (*94*, *95*)] at minimal or similar efficacious doses for relatively short treatment timespans, relative to long term uses in humans, producing less severe effects on lean mass and food intake, as previously observed (*95*, *96*).

#### Mouse diet-induced MASLD/MASH studies

Experiments were performed using 6-week-old male C57BL/6 J mice purchased from The Jackson Laboratory, Bar Harbor, ME. The mice were fed a diet containing L-Amino Acid Diet With 60 kcal% Fat With 0.1% Methionine and No Added Choline (Research Diets A06071302i) for eight weeks before and thereafter during treatment. After reaching study design start for treatment, mice were treated with either vehicle control (5% NMP, 20% PEG-200) or TOFA twice (BID) daily, as described in study designs. Body weight was measured every week. Upon sacrifice, liver was collected and ∼1 mL of blood was obtained from each mouse by cardiac puncture. Blood was centrifuged at 2,000 g for 5 min at 4°C to obtain serum, which was frozen at −80°C. All mouse procedures were approved by the University of California, Berkeley Institutional Animal Care and Use Committee (Protocol # AUP-2018-10-11513-2). All protocols conform to federal regulations, the National Research Council Guide for the Care and Use of Laboratory Animals, and the Public Health Service Policy on Humane Care and Use of Laboratory Animals.

At six weeks of age, *MUP-uPA* mice were fed a 60 kcal% fat diet (Research diets, D12492i) ad libitum. Mice were weighed at regular intervals for weight tracking. Food intake was measured by weighing food amounts remaining in the food hopper at regular intervals. At designated experimental timepoints, compound treatments were administered by oral gavage (PO). All animal studies were performed in accordance with NIH guidelines for the use and care of live animals and approved by University of California, San Diego (UCSD) Institutional Animal Care and Use Committee (Protocol # S00218).

#### Plasma concentration of TOFA in mice over time

Charles River Laboratories was contracted to perform this study via in vivo PK services. Mice were dosed with either 50 mg/kg IP or 250 mg/kg PO TOFA (*n* = 3 per group). Blood samples were collected at various time points for each injection arm: IP (0.0833, 0.250, 0.5, 1, 2, 4, 8, and 24 hours) and PO (0.25, 0.5, 1, 2, 4, 6, 8, and 24 hours). Mass spectrometry (assay specifications, performance, and acceptance criteria defined by company) was utilized to measure TOFA concentration in plasma samples.

#### Tissue sample preparation and gene expression measurement

All tissue samples were rapidly dissected, snap frozen in liquid nitrogen, and stored at −80°C until mRNA and protein extraction. For mRNA quantification, QIAzol (Qiagen) was used for toral RNA extraction from either mouse tissues or cells. The iScript Reverse Transcription Supermix (BioRad) was used for reverse transcription. cDNA was further evaluated by real-time PCR for gene expression using PowerUp SYBR Green (Applied Biosystems) with the QuantStudio6 Real-Time PCR system (Applied Biosystems). The amount of indicated mRNA was normalized to the amount of r18s mRNA. All primer sequences are listed in Table 2.

**Table 2.** Primer sequences used for RT-qPCR, including target gene, primer direction (forward/reverse), and sequence.

Gene Symbol	Species	Forward Oligo (5′-3′)	Reverse Oligo (5′-3′)
*Acot1*	Mouse	ATACCCCCTGTGACTATCCTGA	CAAACACTCACTACCCAACTGT
*Angptl4*	Mouse	GTTTGCAGACTCAGCTCAAGG	CCAAGAGGTCTATCTGGCTCTG
*Atp5g*	Mouse	CCTGTGCCTGTCTTTCTACC	CCTTCCACACTCTGCCTTATC
*Bnip3*	Mouse	GCTCCTGGGTAGAACTGCAC	GCTGGGCATCCAACAGTATT
*Cd36*	Mouse	GAGCAACTGGTGGATGGTTT	GCAGAATCAAGGGAGAGCAC
*Cidea*	Mouse	AGGCCGTGTTAAGGAATCTGC	TAGACCAGGAACTGTCCCGT
*Col1a1*	Mouse	CCGATGGATTCCCGTTCGAG	ACATTAGGCGCAGGAAGGTC
*Col3a*	Mouse	AACCTGGTTTCTTCTCACCCTTC	ACTCATAGGACTGACCAAGGTGG
*CPT1A*	Human	GCAAAGGCGACATCAATCCG	AACCTCTTGACATTCCCCCG
*Cpt1b*	Mouse	GCACACCAGGCAGTAGCTTT	CAGGAGTTGATTCCAGACAGGTA
*Cyp4a14*	Mouse	TTTAGCCCTACAAGGTACTTGGA	GCAGCCACTGCCTTCGTAA
*Drp1*	Mouse	GTTCCACGCCAACAGAATAC	CCTAACCCCCTGAATGAAGT
*Elovl7*	Mouse	ATCTTACATCGAGGACTGTGCG	TAGTCTTCAACTCTCGGATCAGC
*Fabp3*	Mouse	ACCTGGAAGCTAGTGGACAG	TGATGGTAGTAGGCTTGGTCAT
*Fgf21*	Mouse	TGGATCGCCTCACTTTGATCC	CTTCTGAGGCAGACGCAGG
*Fis1*	Mouse	AAGTATGTGCGAGGGCTGT	TGCCTACCAGTCCATCTTTC
*Fundc1*	Mouse	CCCCCTCCCCAAGACTATGAA	CCACCCATTACAATCTGAGTAGC
*IL1b*	Mouse	GCCACCTTTTGACAGTGATGAG	TGCTGCGAGATTTGAAGCTG
*IL6*	Mouse	TAGTCCTTCCTACCCCAATTTCC	TTGGTCCTTAGCCACTCCTTC
*Lpl*	Mouse	GAGGACTCGGAGACGTGGA	TGTATGCCTTGCTGGGGTTT
*Mfn1*	Mouse	GACCCGTGCGAAAGAGAGAG	TCGAGCAAAAGTAGTGGCCA
*Mfn2*	Mouse	ATGTTACCACGGAGCTGGAC	AACTGCTTCTCCGTCTGCAT
*Nrf2*	Mouse	CGGTGGGTCTCCGTAAATGG	TGAACTCCTGGACGGGACTA
*Opa1*	Mouse	ATACTGGGATCTGCTGTTGG	AAGTCAGGCACAATCCACTT
*Pdk4*	Mouse	CCGCTTAGTGAACACTCCTTC	TCTACAAACTCTGACAGGGCTTT
*Pgc1a*	Mouse	TATGGAGTGACATAGAGTGTGCT	CCACTTCAATCCACCCAGAAAG
*Pink1*	Mouse	TGAGGAGCAGACTCCCAGTT	AGTCCCACTCCACAAGGAT
*Prdm16*	Mouse	CTTAGCCGGGAAGTCACAGG	ATTGCATATGCCTCCGGGT
*Prkn*	Mouse	TGGAAAGCTCCGAGTTCAGT	CCTTGTCTGAGGTTGGGTGT
*R18S*	Human	GTAACCCGTTGAACCCCATT	CCATCCAATCGGTAGTAGCG
*r18s*	Mouse	GCAATTATTCCCCATGAACG	GGCCTCACTAAACCATCCAA
*Srebpf1c*	Mouse	GGAGCCATGGATTGCACATT	GGCCCGGGAAGTCACTGT
*Tfam*	Mouse	TGGCAGTCCATAGGCACCGTATT	ACAGACAAGACTGATAGACGAGGG
*Tnfa*	Mouse	CCCTCACACTCAGATCATCTTCT	GCTACGACGTGGGCTACAG
*Ucp1*	Mouse	GGATTGGCCTCTACGACTCA	ACACCTCCAGTCATTAAGCCG
*Ucp2*	Mouse	ACTTCACTTCTGCCTTCGGG	GGAAGGCATGAACCCCTTGT
*Ucp3*	Mouse	CTGCACCGCCAGATGAGTTT	ATCATGGCTTGAAATCGGACC
*VLDLR*	Human	CCTGCCAGCACCACAGATT	TGGTCACATTGATCCTTTGACAG
*Vldlr*	Mouse	CTGGTTCCTGGAGGGATCAAT	CAGGCAGCTGAAGTCCCTTT

#### RNA-sequencing and bioinformatics analysis

cDNA libraries were constructed from tissue-extracted RNA samples according to the manufacturer’s protocol (Roche KAPA HyperPrep kit). Libraries were sequenced on the NovaSeq6000 platform (Novogene), resulting in approximately 25 million reads per sample on average. Transcriptome mapping was performed with STAR using the Ensembl annotation of the mm10 reference genome. Read counts for individual genes were generated using HTSeq (*97*). Differential expression analysis was performed using EdgeR (*98*) after normalizing read counts and including only those genes with CPM > 1 for at least one sample. Differentially expressed genes (DEGs) were defined based on the criteria of >2-fold absolute change in expression value and adjusted *P* value <0.05. Analysis of enriched functional categories among detected genes was performed using Enrichr (*38*) and Gene Set Enrichment Analysis (GSEA) (*43*) using the Molecular Signatures Database (MsigDB) Hallmark gene sets (*99*).

#### Histology

Liver tissues from select animal studies were section for histology. Paraffin-embedded sections were prepared for H&E and Sirius Red staining and the samples were processed in the histology core facility at the University of California, San Francisco Liver Center. Fresh liver sections were prepared for ORO staining. The completed slides were examined and representative images were taken.

#### Metabolic cage housing and energy expenditure measurements

Mice were individually housed and maintained at experimental design housing conditions (12-hour light-dark cycle with pre-designated temperature conditions). The Oxymax-CLAMS system (Columbus Instruments) was used to measure the calorimetry indirectly. Mice were provided ad libitum access to food and water during the entire experimental time course. Oxygen consumption, carbon dioxide production, and energy expenditure were monitored in consecutive light-dark cycles over successive days. Body temperature was measured using a rectal thermometer.

Statistical analysis for metabolic cage data was performed using analysis of covariance (ANCOVA) for body composition-adjusted comparisons (*35*). ANCOVA models included lean mass and fat mass as continuous covariates: EE = β_0_ + β_1_(lean mass) + β_2_(fat mass) + β_3_(treatment) + ε. The assumption of homogeneous regression slopes was verified by testing treatment × covariate interactions. Adjusted means were calculated at the grand mean of covariates across all animals. All analyses were performed in R (v4.3.0) using the ‘car’ and ‘emmeans’ packages.

#### Oxygen consumption measurement (seahorse assay)

Cells (human hepatocellular cells (HepG2) or primary mouse hepatocytes) were plated in poly-D-lysine- or collagen-coated, respectively, XF24-well cell culture microplates (Agilent) before treatment with respective compounds. Oxygen consumption and mitochondrial function was measured by XF24 Extracellular Flux Analyzer (Agilent) using the Seahorse XF Cell Mito Stress Test Kit (Agilent) according to manufacturer’s protocol.

#### Intraperitoneal glucose tolerance test and insulin tolerance test

Mice were fasted 6 hours (GTT) or 2 hours (ITT) during the light phase before the tests. Fasted blood samples were collected by tail vein in Z-clot activator tubes (Sarstedt) for processing to plasma and subsequent metabolite analysis. For glucose tolerance test, 1 g/kg Dextrose (Fisher Scientific) was injected intra-peritoneally into the mice and the glucose levels were measured at 0 (before injection), 15, 30, 60, 90, and 120 minutes post-injection by a glucometer (Bayer ContourNext). For insulin tolerance test, 0.5 U/kg insulin (Lilly) was injected intra-peritoneally into the mice and the glucose levels were measured at 0 (before injection), 15, 30, 60, 90, and 120 minutes post-injection by a glucometer (Bayer ContourNext). To assess area of the curve (AOC) for GTT and ITT, area under the baseline of starting blood glucose levels was subtracted from area under the curve (AUC) values to account for differences in starting blood glucose levels as previously described (*100*).

#### Treadmill run to endurance

Prior to exhaustion running, mice were pre-adapted to the treadmill (Columbus Instruments) for 10 minutes per day for 2 days at a gradually increased speed (5 to 10 meter/min). The same treadmill was used for the run-to-exhaustion test, which included 10 minutes of adaptive period with a gradually increasing speed from 5 to 12 m/min followed by increases of 1 m/min every 10 minutes up to 15 m/min followed by increases of 1 m/min every 5 minutes up to 20 m/min until mice failed.

#### Fecal bomb calorimetry assay

Male C57BL/6 J wild-type mice were maintained on a 60 kcal% fat diet (Research diets, D12492i) ad libitum) for two weeks prior to treatment. Mice were then administered a single dose of either vehicle control or TOFA (250 mg/kg) via oral gavage. Beginning 24 hours after the first administration (t = 0 hours), mice were single-housed and received a second oral gavage dose. Fecal samples were collected at the end of the 48-hour single-housing period (t = 48 h) and stored appropriately prior to analysis. All mouse procedures were approved by the University of California, Berkeley Institutional Animal Care and Use Committee (Protocol # AUP-2018-10-11513-2). All protocols conform to federal regulations, the National Research Council Guide for the Care and Use of Laboratory Animals, and the Public Health Service Policy on Humane Care and Use of Laboratory Animals. Gross energy content of fecal samples was determined by bomb calorimetry at the University of Michigan Animal Phenotyping Core. Fecal samples were dried to a constant weight, ground, and pelleted with wheat flour of pre-measured energy content. Dried samples were combusted in pure oxygen using a Parr 6200 bomb calorimeter equipped with an 1108P oxygen bomb, and heat production was recorded to calculate gross caloric content (cal/g).

#### Biochemical assays

Assays for measurement of tissue and serum metabolites (triglycerides, cholesterol, ALT, AST, urea, insulin, FGF-21, hydrogen peroxide) were measured by assay kits according to manufacturer’s protocol.

#### ACC activity assays

ACC1 (BPS #79315) and ACC2 (BPS #79282) activity assay were used to measure respective ACC activity levels of TOFA at a top dose of 1 mM with a 5-fold serial dilution for 8 concentrations (performed in duplicates). TOFyl-CoA was tested at a top dose of 500 μM with a 10-fold serial dilution for 8 concentrations (performed in duplicates). Firsocostat was used as a positive control to ensure assay responsiveness from a top dose of 10 μM with a 10-fold serial dilution for 9 concentrations (performed in duplicates). ADP-Glo Kinase assay was used as a complementary readout for ACC activity (Promega V6930). Assay was carried out according to manufacturer’s instructions.

#### PPAR thermal shift assays

*The* thermal shift assay was performed using the Applied Biosystems Protein Thermal Shift kit (Thermo Fisher Scientific, 4461146) according to the manufacturer’s instructions. PPARα protein ligand binding domain (amino acids 200–468 with N-terminal 6xHis-tag) was expressed and purified as previously described (*77*). For each replicate, 2.7 μg of the PPARα protein ligand binding or 2 μg of the PPARδ ligand binding domain (Thermo Fisher Scientific, PV4694) was used. TOFA was tested at various different concentrations: 20 μM, 10 μM, 5 μM, 2 μM, 1 μM and 0.1 μM. Protein melt reactions were carried out using the QuantStudio 6 Pro Real-Time PCR System, and data analysis was performed using the ΔTm-Derivative method as recommended by the manufacturer.

#### Nuclear hormone receptor screening panel

Eurofins DiscoverX was contracted to profile one compound (TOFA) with 36 selected biosensor assays (Eurofin Item #86-0117DR). The assays range from a protein interaction or nuclear translocation format to monitor the activation of a nuclear hormone receptor. In the protein interaction assay, detection is based on protein-protein interactions between an activated, full length human NHR protein and nuclear fusion protein containing co-activator domains with one or more interaction motifs. In the nuclear translocation assay, the assay monitors movement of a human NHR between the cytoplasmic and nuclear compartments. TOFA was evaluated at a top dose of 10 μM with a 3-fold serial dilution curve for 10 concentrations. Protocol was carried out based on manufacturer’s instructions.
