# Supplementary material for: A multi-functional oral small molecule targeting energy and lipid metabolism to treat obesity and related metabolic disorders
Source: Sci Adv. 2026 Aug 21;12(34):eaed3119. doi: 10.1126/sciadv.aed3119 (PMC13496183; doi:10.1126/sciadv.aed3119)
Supplement: Supplementary file 1 [file sciadv.aed3119_sm.pdf]

Science Advances
AAAS

Supplementary Materials for

A multi-functional oral small molecule targeting energy and lipid metabolism to treat obesity and related metabolic disorders

Justin Y. Lee *et al.*

Corresponding author: Anders M. Näär, naar@berkeley.edu

Sci. Adv. **12**, eaed3119 (2026)
DOI: 10.1126/sciadv.aed3119

This PDF file includes:

Figs. S1 to S12
Tables S1 and S2

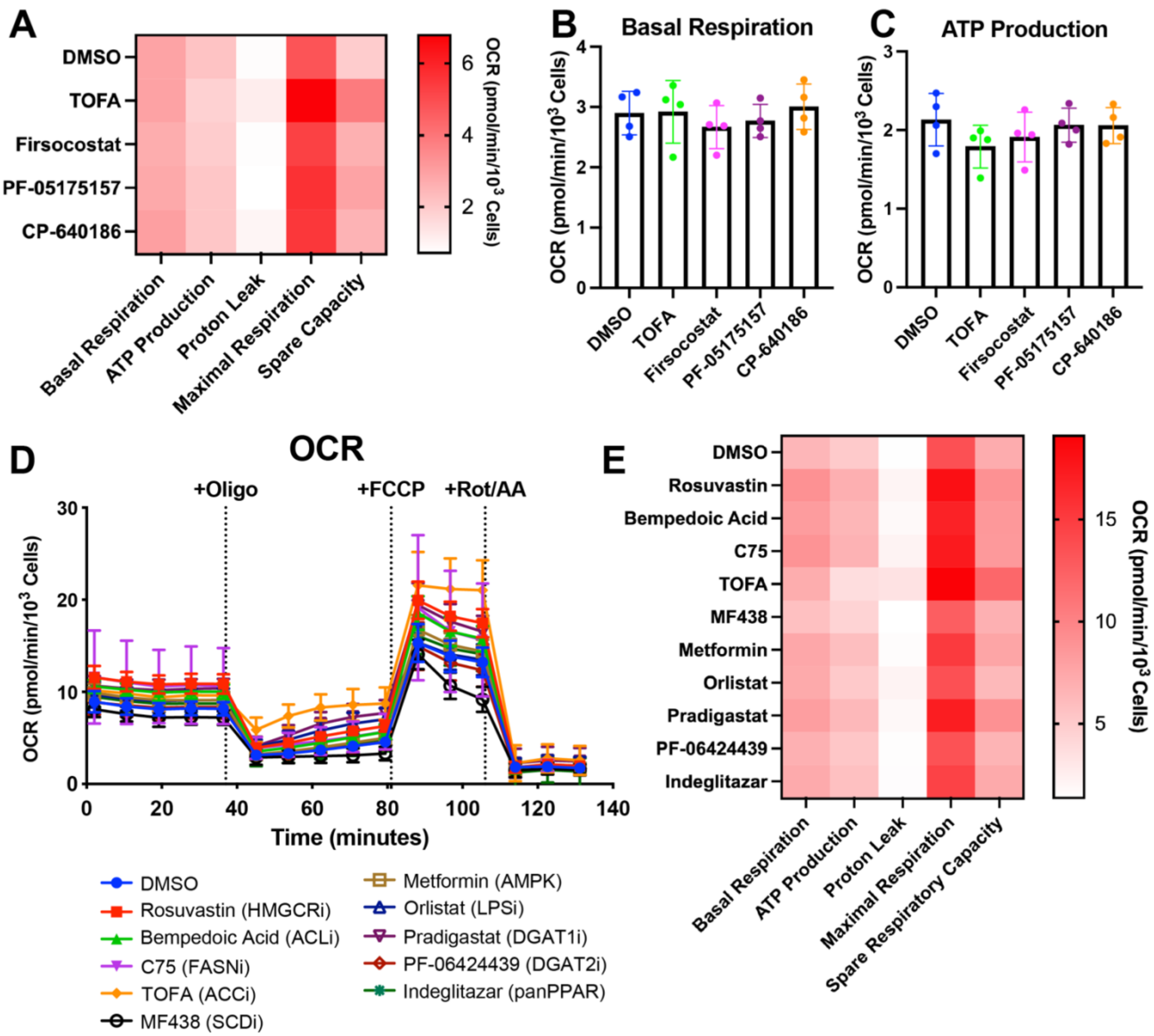

Figure S1. TOFA treatment demonstrates differentiated mitochondrial functional outcomes compared to other lipogenesis targeting small molecules.

From Figure 1D-G, **(A)** heat map representation of area under the curve calculations for various respiration parameters (basal respiration, ATP production, proton leak, maximal respiration, spare respiratory capacity). Individual plots of select respiration parameters: **(B)** basal respiration, and **(C)** ATP production.

(D – E) HepG2 cells were seeded in the Seahorse XF plate, treated with 10 µM of various lipogenesis targeting compounds (maximal inhibitory or effective doses; $IC_{50}/EC_{50} < 10$ µM), and processed through the mitochondrial stress test.

- **(D)** Oxygen consumption rate plotted during treatment time course; n=6/group.
- **(E)** Heat map representation of area under the curve calculations for various respiration parameters (basal respiration, ATP production, proton leak, maximal respiration, spare respiratory capacity).

Data are presented as mean $\pm$ SD.

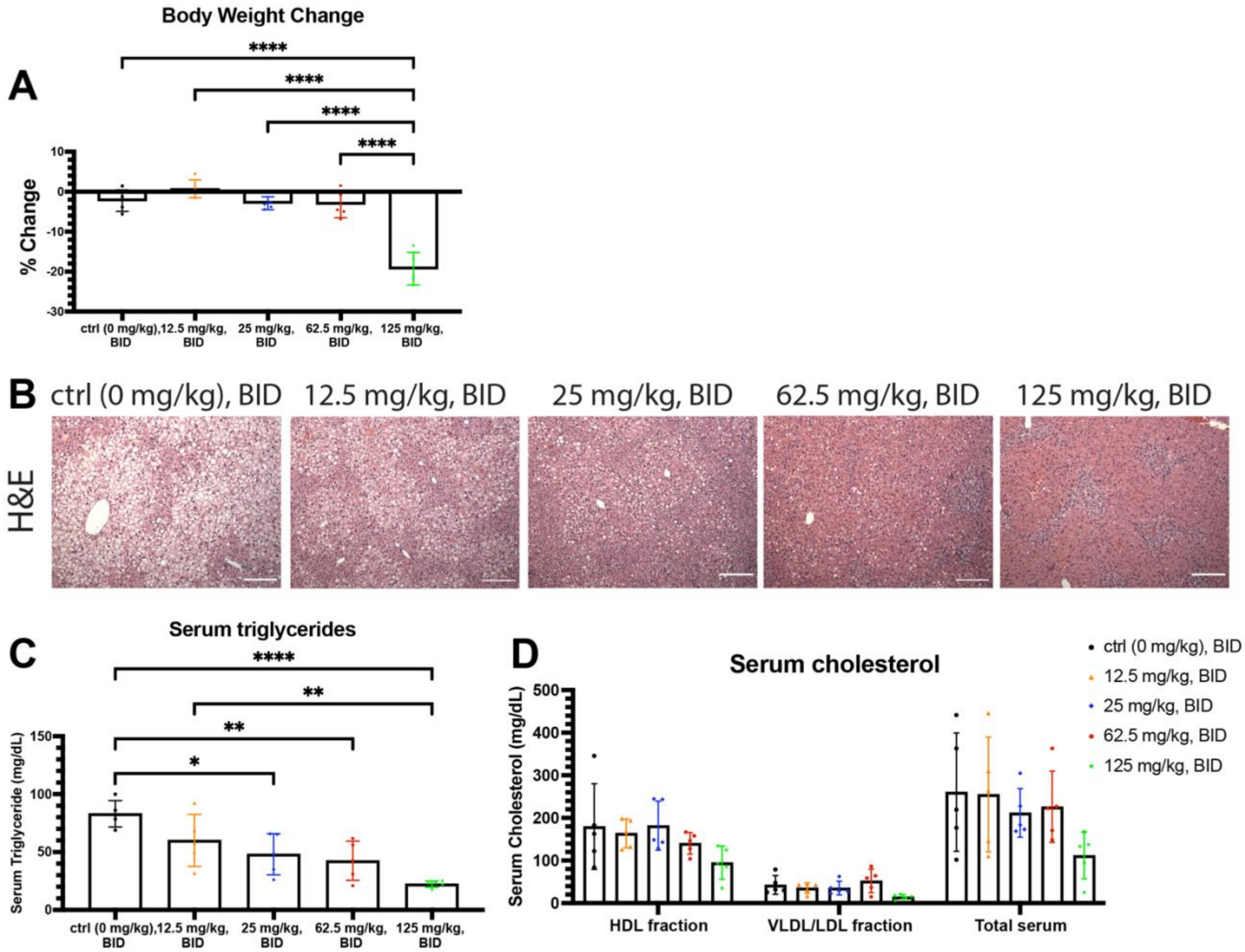

Figure S2. TOFA treatment improves metabolic status in a diet-induced mouse model of obesity and metabolic syndrome in a dose-dependent manner.

(A – D) Male C57BL/6J mice were placed on a 60% HFD for ten weeks and treated for two weeks with vehicle or TOFA at varying doses, BID, as indicated; n = 5/group.

- **(A)** Body weight change.
- **(B)** Representative H&E staining of liver sections. Scale bars, 200 µM (inset).
- **(C)** Serum triglycerides.
- **(D)** Serum total, HDL, and VLDL/LDL cholesterol fractions.

Data are presented as mean $\pm$ SD. *$p < 0.05$, **$p < 0.01$, ***$p < 0.001$, ****$p < 0.0001$ (one way ANOVA).

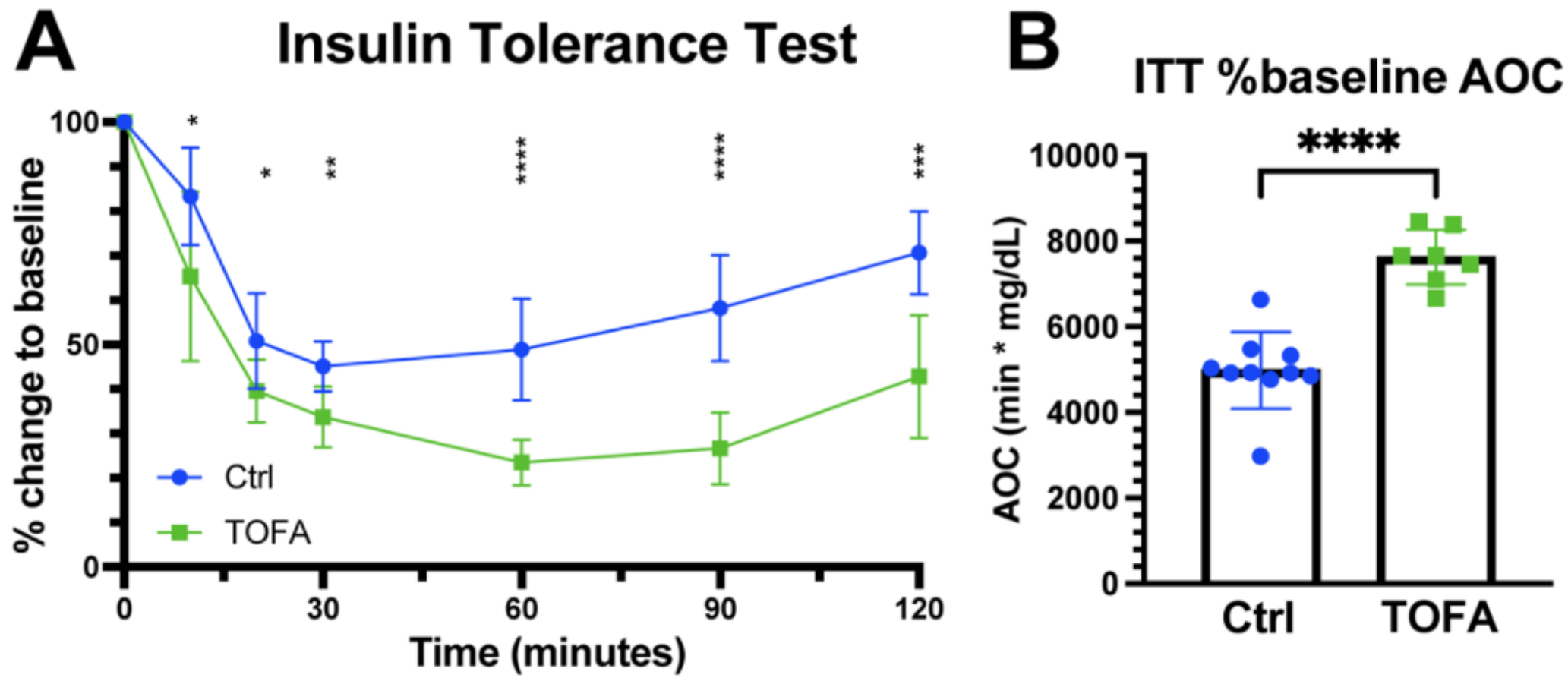

Figure S3. Insulin tolerance tests relative changes to starting baseline blood glucose levels. From Figure 2F-G, tracking of blood glucose level percent changes from baseline reading levels for **(A)** insulin tolerance test, and **(B)** associated area of the curve (AOC) calculation; ****p<0.0001. Data are presented as mean + SD (unpaired two-tailed t test).

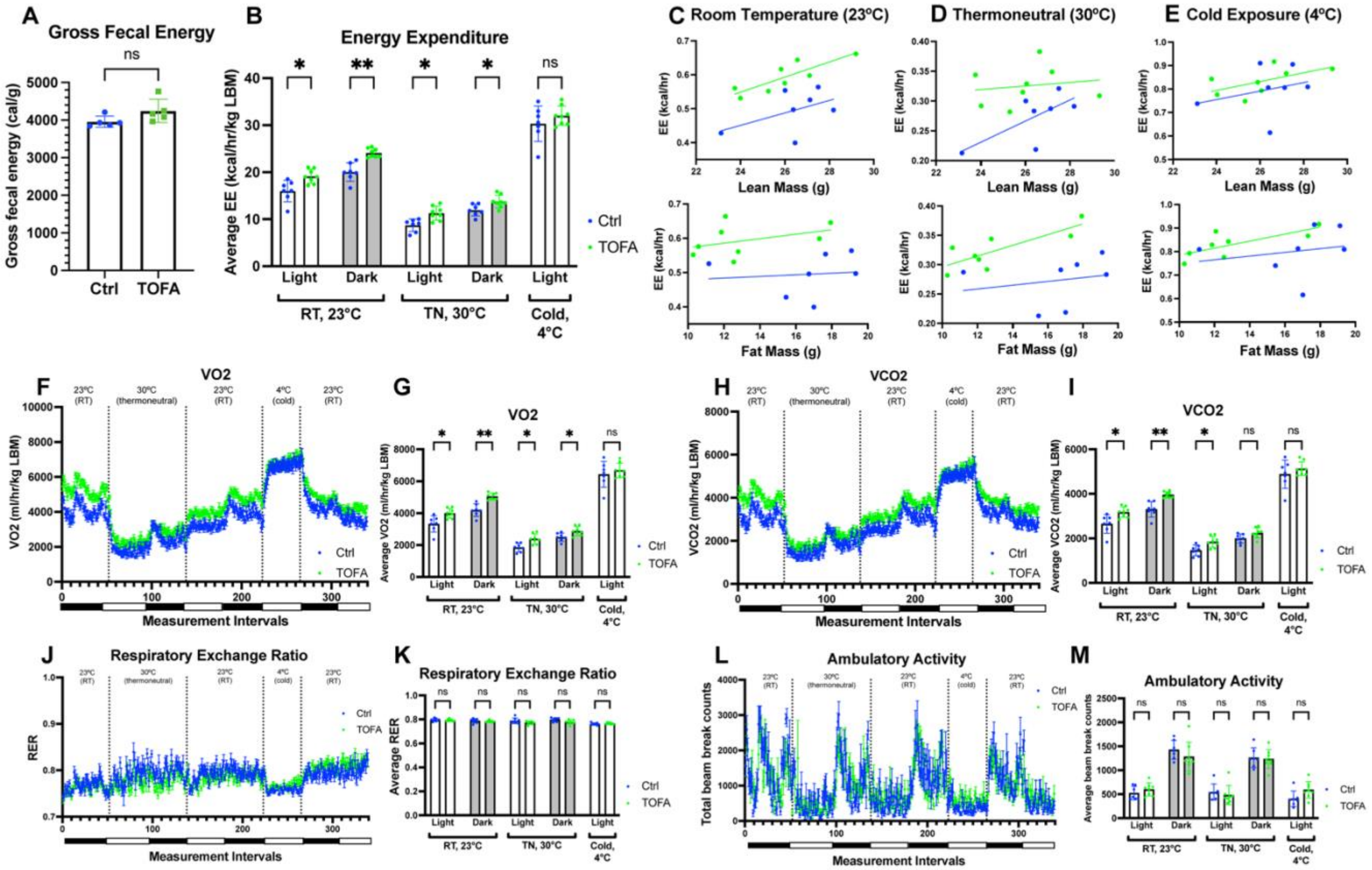

Figure S4. Energy expenditure assessment by calorimetry with metabolic cage analysis using ANCOVA using body mass as a covariate.

- **(A)** Fecal matter from male C57BL/6J mice placed on a 60% HFD and orally treated with vehicle or TOFA (250 mg/kg) once daily was collected after 48 hours and gross caloric content was assessed by bomb calorimetry; p=0.098 (ns); n=5/group. Additional caloric content parameters are provided in Supplementary Table 1.

From Figure 3B, additional metabolic cage parameters were assessed and analyzed.

- **(B)** Total average energy expenditure across light-dark cycles and temperature conditions (RT- room temperature, TN- thermoneutral); *p=0.032, **p=0.0042, *p=0.0138, *p=0.033, p=0.315 (ns).

(C – E) Regression plots of average energy expenditure (EE) as a function of lean mass (top) and fat mass (bottom) for each metabolic cage temperature environment. **(C)** Room temperature (23°C) **(D)** Thermoneutral (30°C) **(E)** Cold Exposure (4°C) Statistical analysis was performed using GLM ANCOVA (Supplementary Table 2).

- **(F)** Oxygen consumption volume and associated **(G)** average VO2 across light-dark cycles and temperature conditions; *p=0.04, **p=0.0032, *p=0.0135, *p=0.04, p=0.485 (ns).
- **(H)** Carbon dioxide production volume and associated **(I)** average VCO2 across light-dark cycles and temperature conditions; *p=0.046, **p=0.0088, *p=0.038, p=0.089 (ns), p=0.373 (ns).
- **(J)** Respiratory exchange ratio and associated **(K)** average RER across light-dark cycles and temperature conditions; p=0.82 (ns), p=0.82 (ns), p=0.40 (ns), p=0.27 (ns), p=0.69 (ns).
- **(L)** Ambulatory activity and associated **(M)** average ambulatory activity levels across light-dark cycles and temperature conditions; p=0.74 (ns), p=0.74 (ns), p=0.77 (ns), p=0.80 (ns), p=0.24 (ns).

Data are presented as mean ± SD. ns- not significant, *$p < 0.05$, **$p < 0.01$, ***$p < 0.001$, ****$p < 0.0001$ (unpaired two-tailed t test, unless otherwise indicated).

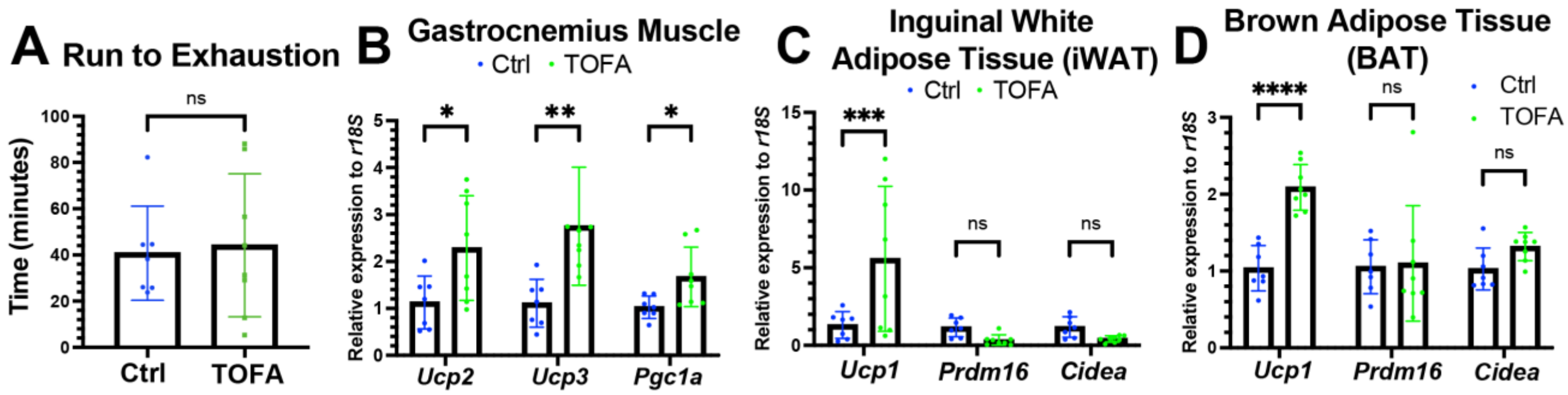

Figure S5. TOFA confers extrahepatic energy expenditure programs. From Figure 3, male C57BL/6J mice were placed on a 60% HFD for eight to twelve weeks and treated for three to four weeks with vehicle or TOFA (200 mg/kg/day), OD; n = 7-8/group.

(A) Run to exhaustion treadmill test run times; p=0.804 (ns).

(B – D) RT-qPCR of mitochondrial function genes in gastrocnemius muscle, inguinal white adipose tissue (iWAT), or brown adipose tissue (BAT), respectively.

Data are presented as mean ± SD. ns- not significant, *p < 0.05, **p < 0.01, ***p < 0.001, ****p < 0.0001 (unpaired two tail t-test).

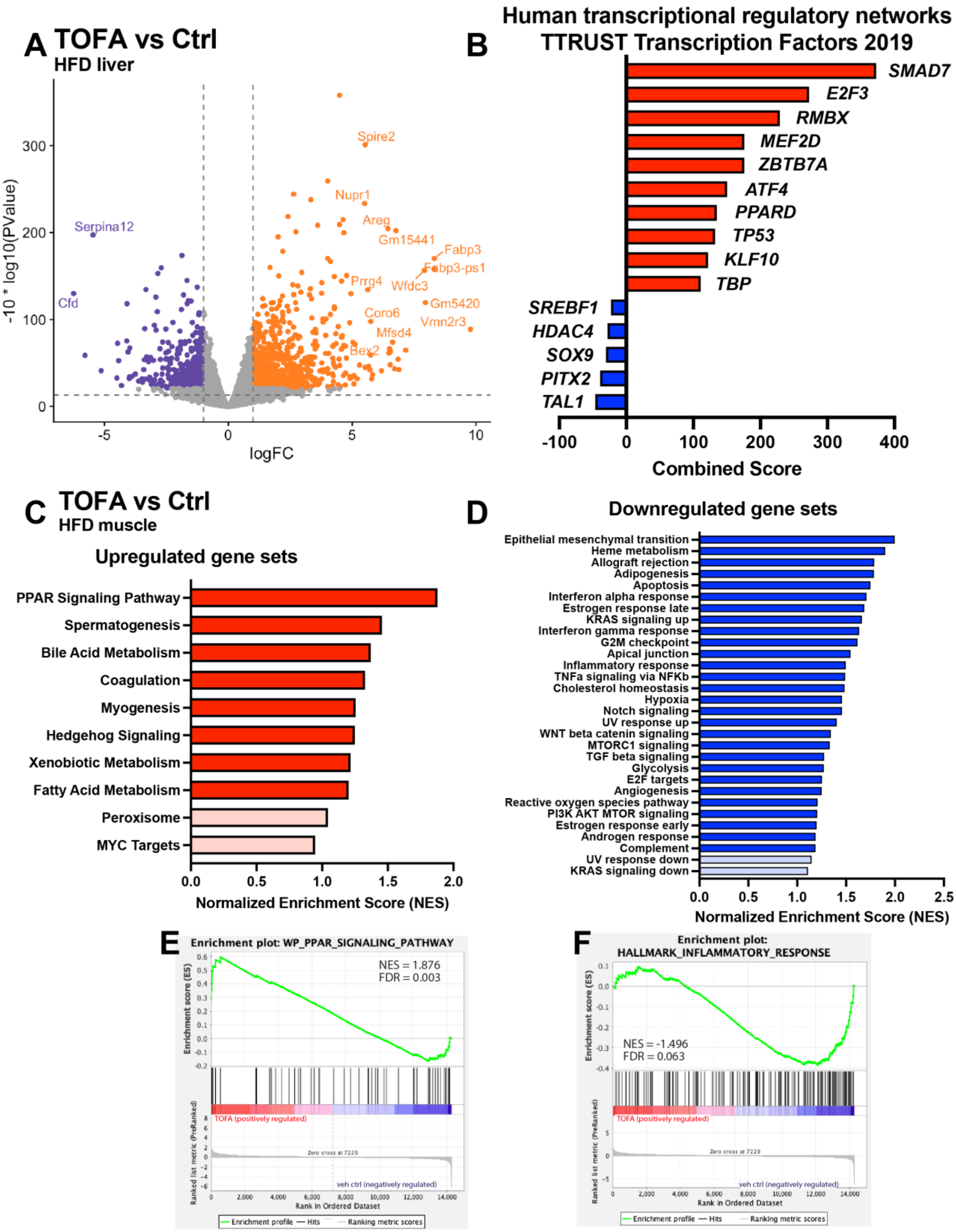

Figure S6. RNA-sequencing of diet-induced obese mice revealed an upregulated PPAR pathway transcriptional signature in liver and skeletal muscle tissue in response to TOFA treatment.

From Figure 4, liver and muscle tissue of mice placed on a 60% HFD for seven weeks and treated for four weeks with vehicle or TOFA were isolated, extracted for RNA, and subjected to RNA sequencing.

- **(A)** Volcano plot of differentially expressed genes (DEGs) in mouse liver samples with a false discovery rate cutoff (FDR) of 0.05 and a fold-change cutoff of 2.
- **(B)** Top upregulated (combined score > 100 and p<0.01) and downregulated (combined score < -20) human transcriptional regulatory networks from the TTRUST Transcription Factors 2019, according to Enrichr analysis(*38*).
- **(C)** Top upregulated Hallmark gene sets by gene set enrichment analysis (GSEA)(*43*) in muscle samples with a normalized enrichment score (NES) cutoff of greater than 0.9. Darker red bars represent false discovery rate (FDR) < 0.25. The PPAR signaling pathway has a p < 0.01.
- **(D)** Top downregulated Hallmark gene sets by GSEA in muscle samples with a normalized enrichment score (NES) cutoff of greater than 1. Darker blue bars represent false discovery rate (FDR) < 0.25. The inflammatory pathway has a p = 0.015.
- **(E)** GSEA of muscle samples focusing on PPAR signaling pathway genes.
- **(F)** GSEA of muscle samples focusing on inflammatory response genes.

Combined score is computed by taking the log of the p-value from the Fisher exact test and multiplying that by the z-score of the deviation from the expected rank. Data are presented as mean ± SD. *p < 0.05, **p < 0.01, ***p < 0.001, ****p < 0.0001 (unpaired two tail t-test).

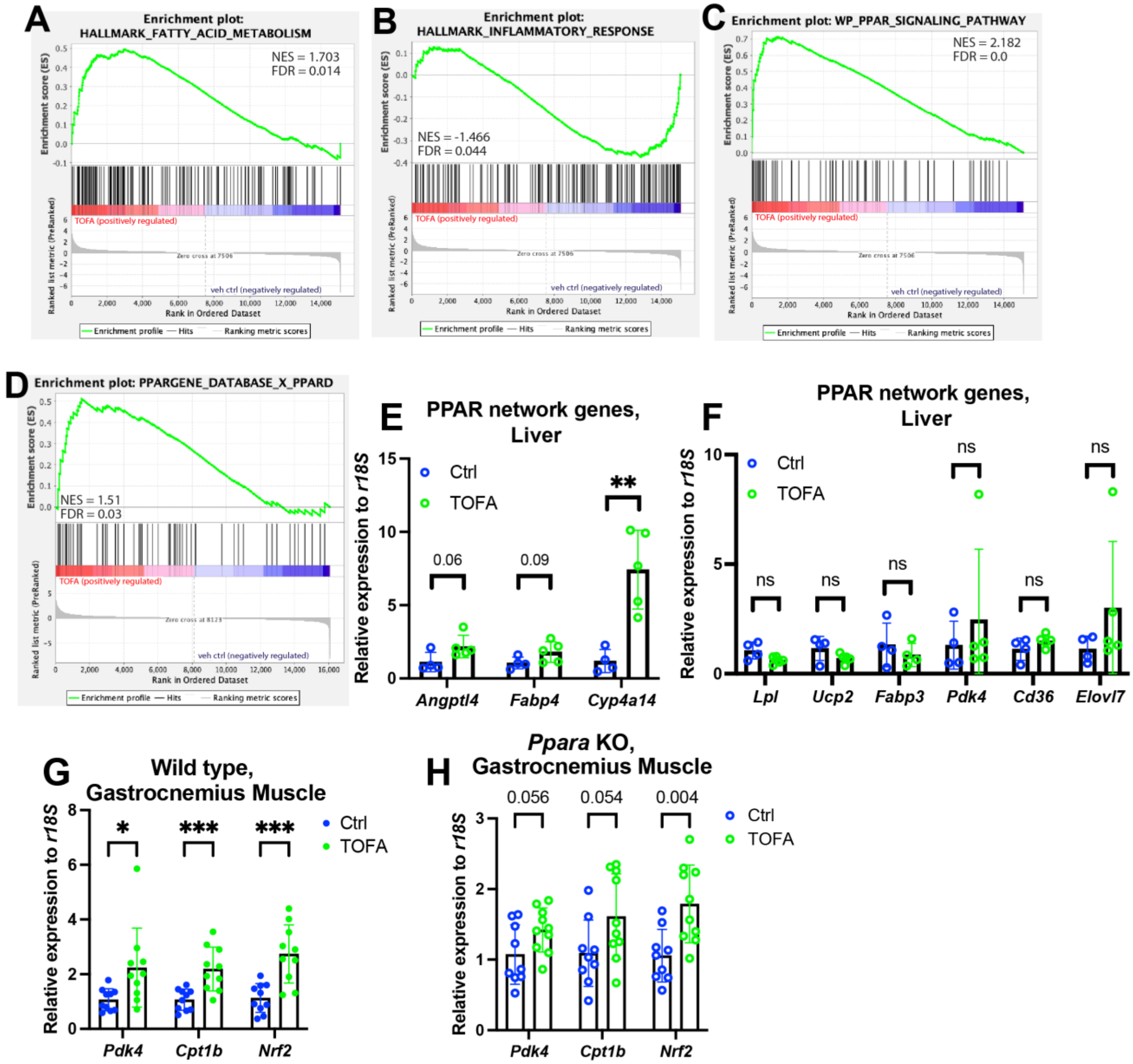

Figure S7. RNA-sequencing from obese whole body *PPARa* knockout mice treated with TOFA revealed a persisting, but modulated, PPAR transcriptional response.

From Figure 5, *Ppara* KO (aKO) mice were placed on a 60% HFD for ten weeks and treated for two weeks with vehicle or TOFA. Liver tissue from *Ppara* KO mice were collected, extracted for RNA, and subjected to RNA sequencing and RT-qPCR.

- **(A)** Gene set enrichment analysis (GSEA)(*43*) focusing on hallmark fatty acid metabolism pathway genes, p < 0.001.
- **(B)** GSEA focusing on hallmark inflammatory response genes, p < 0.001.
- **(C)** GSEA focusing on PPAR signaling pathway genes, p < 0.001.
- **(D)** GSEA focusing on *Ppard* genes from the PPARGENE Database, p = 0.087.
- **(E)** RT-qPCR in mouse liver of PPAR network genes, grouped by changed gene expression; n=4-5/group; **p=0.00477.

(F) RT-qPCR in mouse liver of PPAR network genes, grouped by no changes in gene expression; n=4-5/group.

(G) RT-qPCR of PPAR network genes in wild-type mouse gastrocnemius muscle fed HFD treated with or without TOFA; n = 10/group; *p=0.0236, ***p=000886, ***p=0.000442.

(H) RT-qPCR of PPAR network genes in *PPARa* KO mouse gastrocnemius muscle fed HFD treated with or without TOFA; n = 9-10/group.

Data are presented as mean ± SD. *p < 0.05, **p < 0.01, ***p < 0.001, ****p < 0.0001 (unpaired two tail t-test).

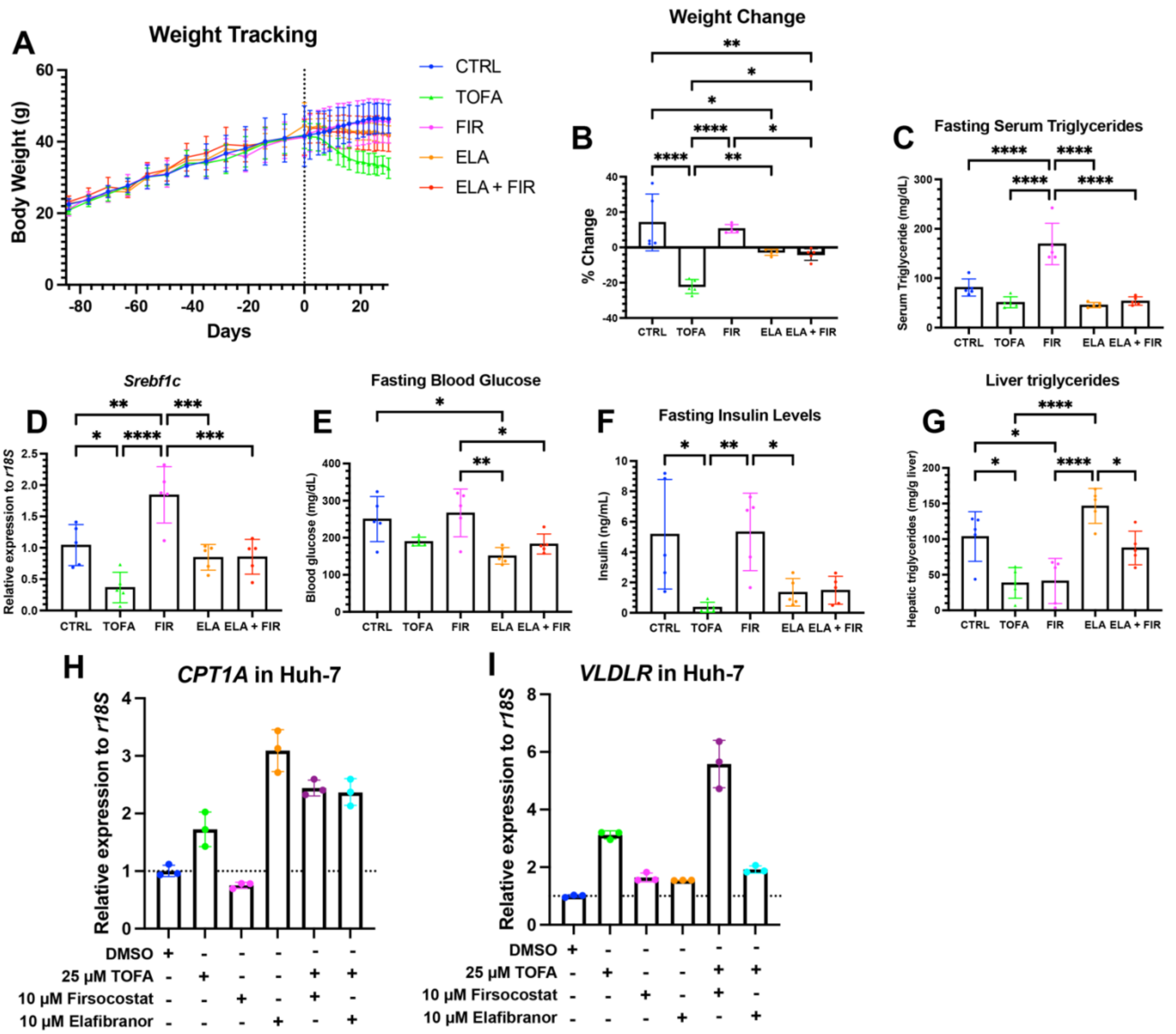

Figure S8. TOFA couples the beneficial actions of ACC inhibition and PPARα+δ agonism in a synergistic manner compared to single targeting compounds with single and combination treatments in diet-induced obese mice.

(A – G) Male C57BL/6J mice were placed on a 60% HFD for twelve weeks and orally treated once daily for 4 weeks with vehicle, TOFA (200 mg/kg/day), Firsocostat (10 mg/kg/day), Elafibranor (10 mg/kg/day), or combination of Elafibranor and Firsocostat; n=5/group.

- **(A)** Body weight
- **(B)** Percent body weight change
- **(C)** Fasting serum triglycerides
- **(D)** RT-qPCR of hepatic *Srebf1c* mRNA transcripts
- **(E)** Fasting blood glucose levels
- **(F)** Fasting blood insulin levels
- **(G)** Liver triglycerides

(H – I) Huh-7 cells were treated with either DMSO, 25 µM TOFA, 10 µM Firsocostat, 10 µM Elafibranor, or co-administered multiple compounds, as denoted in figure, for 24 hours then processed for gene expression analysis by RT-qPCR of **(H)** *CPT1A* and **(I)** *VLDLR*; n=3/group.

Data are presented as mean $\pm$ SD. *$p < 0.05$, **$p < 0.01$, ***$p < 0.001$, ****$p < 0.0001$ (one way ANOVA).

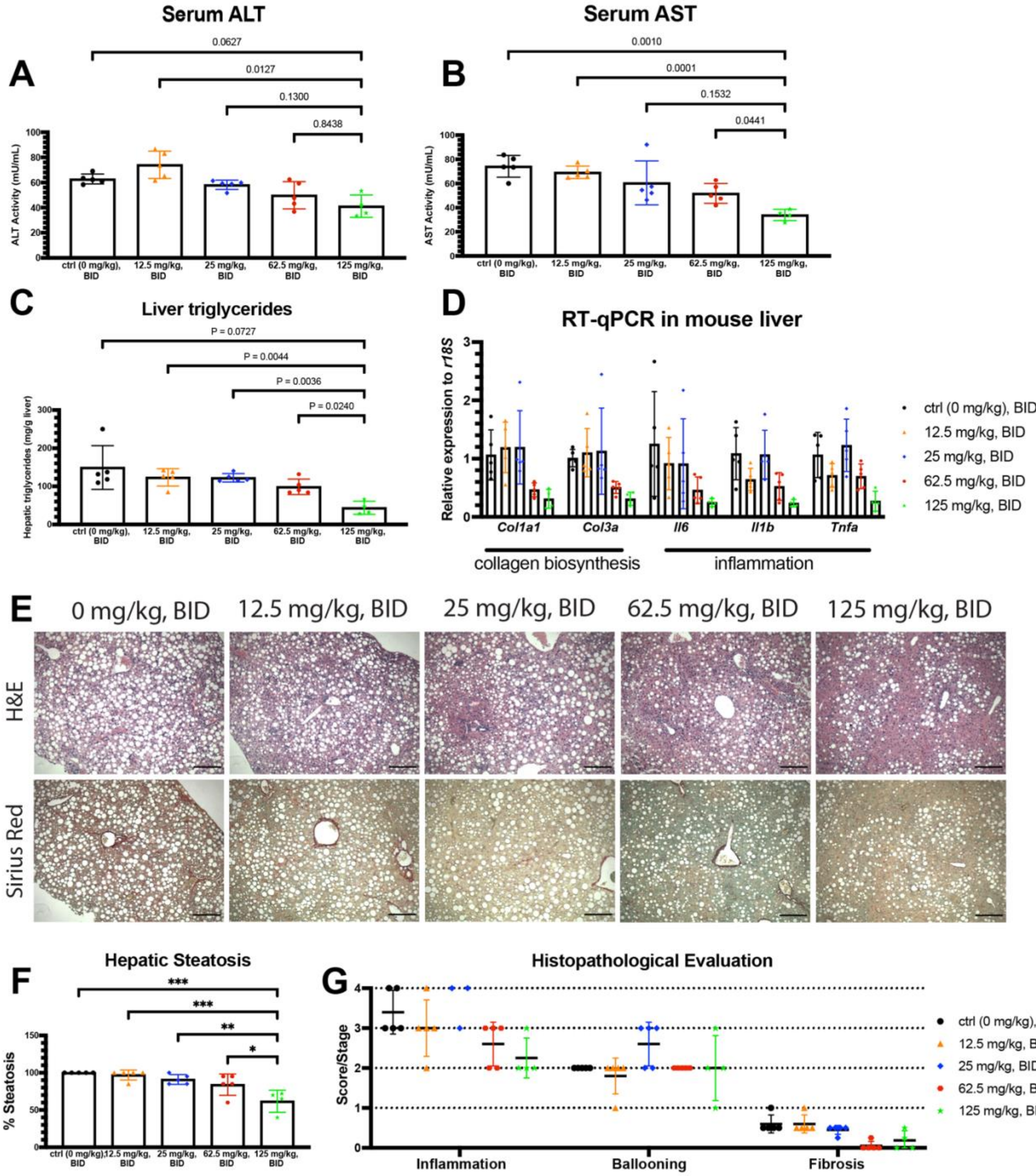

Figure S9. TOFA treatment counters pathological features of MASLD/MASH in the CDAA-HFD mouse model in a dose-dependent manner.

(A – G) Male C57BL/6J mice were placed on a CDAA-HFD for three weeks and treated for three weeks with vehicle or TOFA at varying doses, BID, as indicated; n = 4-5/group.

- **(A)** Serum ALT
- **(B)** Serum AST
- **(C)** Liver triglycerides
- **(D)** RT-qPCR in mouse liver of collagen biosynthesis and inflammation genes.

- **(E)** Representative H&E and Sirius Red staining of liver sections. Scale bars 200 µM (inset).
- **(F)** Hepatic steatosis
- **(G)** MASH activity scores

Data are presented as mean ± SD. *p < 0.05, **p < 0.01, ***p < 0.001, ****p < 0.0001 (one way ANOVA).

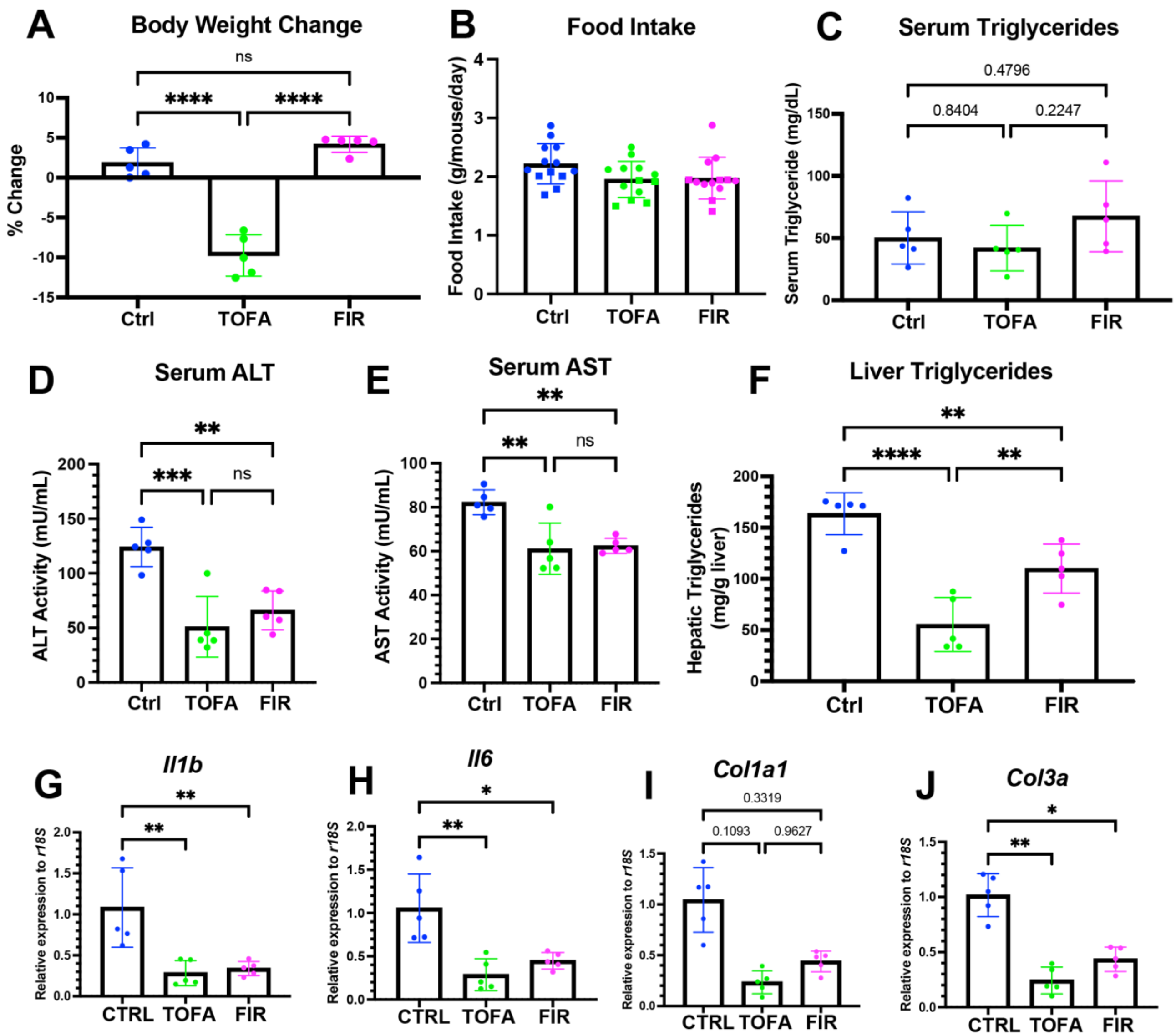

Figure S10. TOFA outperforms Firsocostat in hepatic improvements in the CDAA-HFD mouse model of dietary-induced MASLD/MASH.

(A – J) Male C57BL/6J mice were placed on a CDAA-HFD for eight weeks and orally treated for four weeks with vehicle, TOFA (250 mg/kg/day), or Firsocostat (FIR) (5 mg/kg/day); n = 5/group.

- **(A)** Body weight change
- **(B)** Food intake
- **(C)** Serum TG
- **(D)** Serum ALT
- **(E)** Serum AST
- **(F)** Liver triglyceride
- **(G)** RT-qPCR of hepatic *Il1b* mRNA transcripts.
- **(H)** RT-qPCR of hepatic *Il6* mRNA transcripts.
- **(I)** RT-qPCR of hepatic *Col1a1* mRNA transcripts.
- **(J)** RT-qPCR of hepatic *Col3a* mRNA transcripts.

Data are presented as mean ± SD. ns, not significant; *p < 0.05, **p < 0.01, ***p < 0.001, ****p < 0.0001 (one way ANOVA).

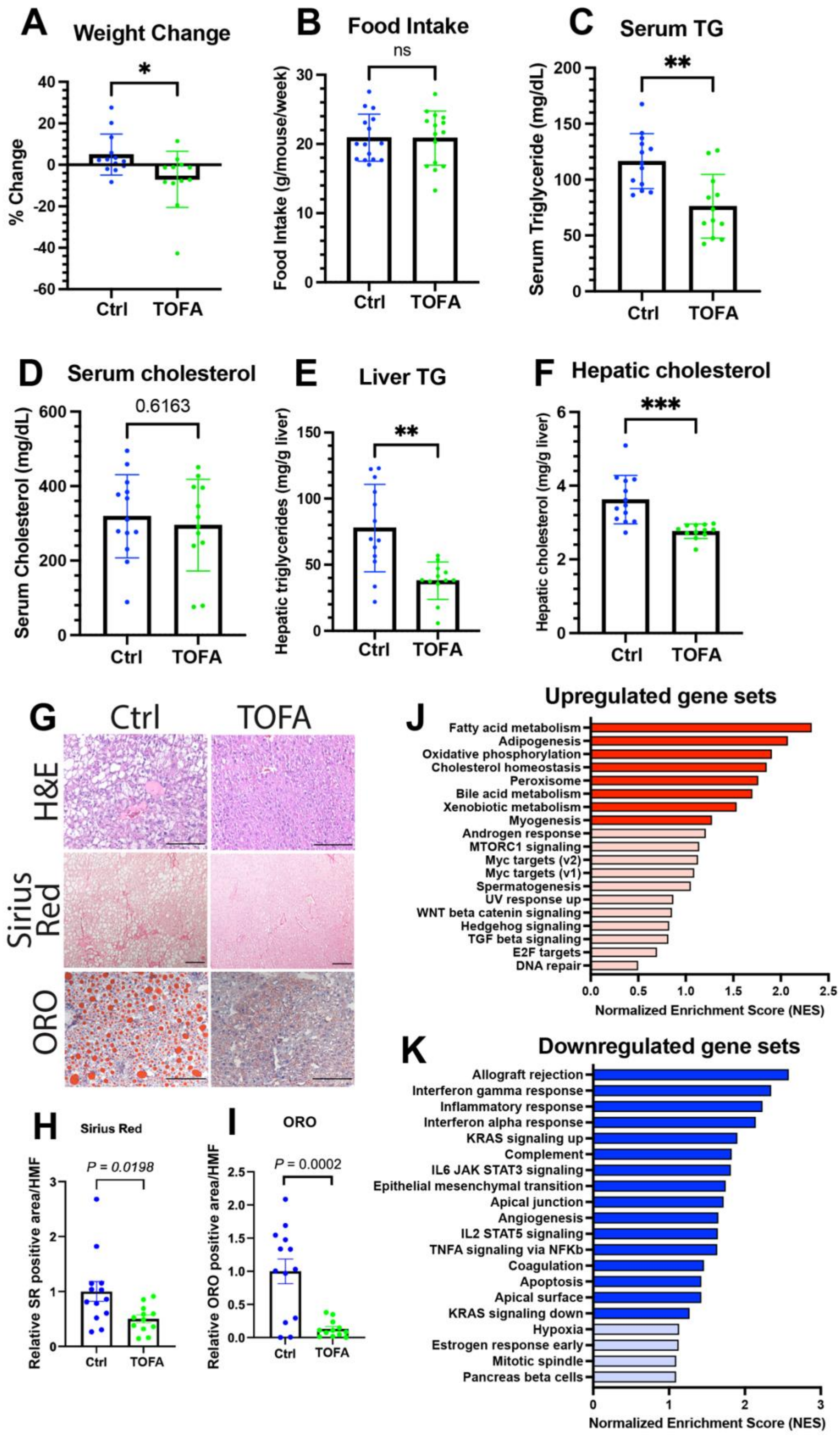

A
Weight Change
% Change
Ctrl
TOFA
B
Food Intake
ns
Food Intake (g/mouse/week)
C
Serum TG
Serum Triglyceride (mg/dL)
D
Serum cholesterol
0.6163
Serum Cholesterol (mg/dL)
E
Liver TG
Hepatic triglycerides (mg/g liver)
F
Hepatic cholesterol
Hepatic cholesterol (mg/g liver)
G
H&E
Sirius Red
ORO
H
Sirius Red
P = 0.0198
Relative SR positive area/HMF
I
ORO
P = 0.0002
Relative ORO positive area/HMF
J
Upregulated gene sets
Fatty acid metabolism
Adipogenesis
Oxidative phosphorylation
Cholesterol homeostasis
Peroxisome
Bile acid metabolism
Xenobiotic metabolism
Myogenesis
Androgen response
MTORC1 signaling
Myc targets (v2)
Myc targets (v1)
Spermatogenesis
UV response up
WNT beta catenin signaling
Hedgehog signaling
TGF beta signaling
E2F targets
DNA repair
Normalized Enrichment Score (NES)
K
Downregulated gene sets
Allograft rejection
Interferon gamma response
Inflammatory response
Interferon alpha response
KRAS signaling up
Complement
IL6 JAK STAT3 signaling
Epithelial mesenchymal transition
Apical junction
Angiogenesis
IL2 STAT5 signaling
TNFA signaling via NFKb
Coagulation
Apoptosis
Apical surface
KRAS signaling down
Hypoxia
Estrogen response early
Mitotic spindle
Pancreas beta cells
Normalized Enrichment Score (NES)

Figure S11. TOFA improves MASH pathology in the *MUP-uPA* on HFD mouse model of MASH.
(A – K) Male *MUP-uPA* mice were placed on a 60% HFD for ten weeks and orally treated for six weeks with vehicle or TOFA (125 mg/kg, BID); n = 12-13/group.

- **(A)** Body weight change; *p=0.0212.
- **(B)** Food intake; p=0.9635 (ns).
- **(C)** Serum triglycerides; **p=0.001.
- **(D)** Serum cholesterol.
- **(E)** Liver triglycerides; **p=0.001.
- **(F)** Liver cholesterol; ***p=0.0005.
- **(G)** Representative H&E, Sirius Red, and Oil Red O (ORO) staining of liver sections. Scale bars, 200 µM (inset).
- **(H)** Sirius Red staining quantification
- **(I)** Oil Red O staining quantification
- **(J)** Gene set enrichment analysis (GSEA)(*43*) of hallmark gene sets upregulated in livers of mouse subjects. Darker red bars represent false discovery rate (FDR) < 0.25.
- **(K)** GSEA of hallmark gene sets downregulated in livers of mouse subjects. Darker blue bars represent false discovery rate (FDR) < 0.25.

Data are presented as mean $\pm$ SD. ns, not significant; *p < 0.05, **p < 0.01, ***p < 0.001. (unpaired two-tailed t test).

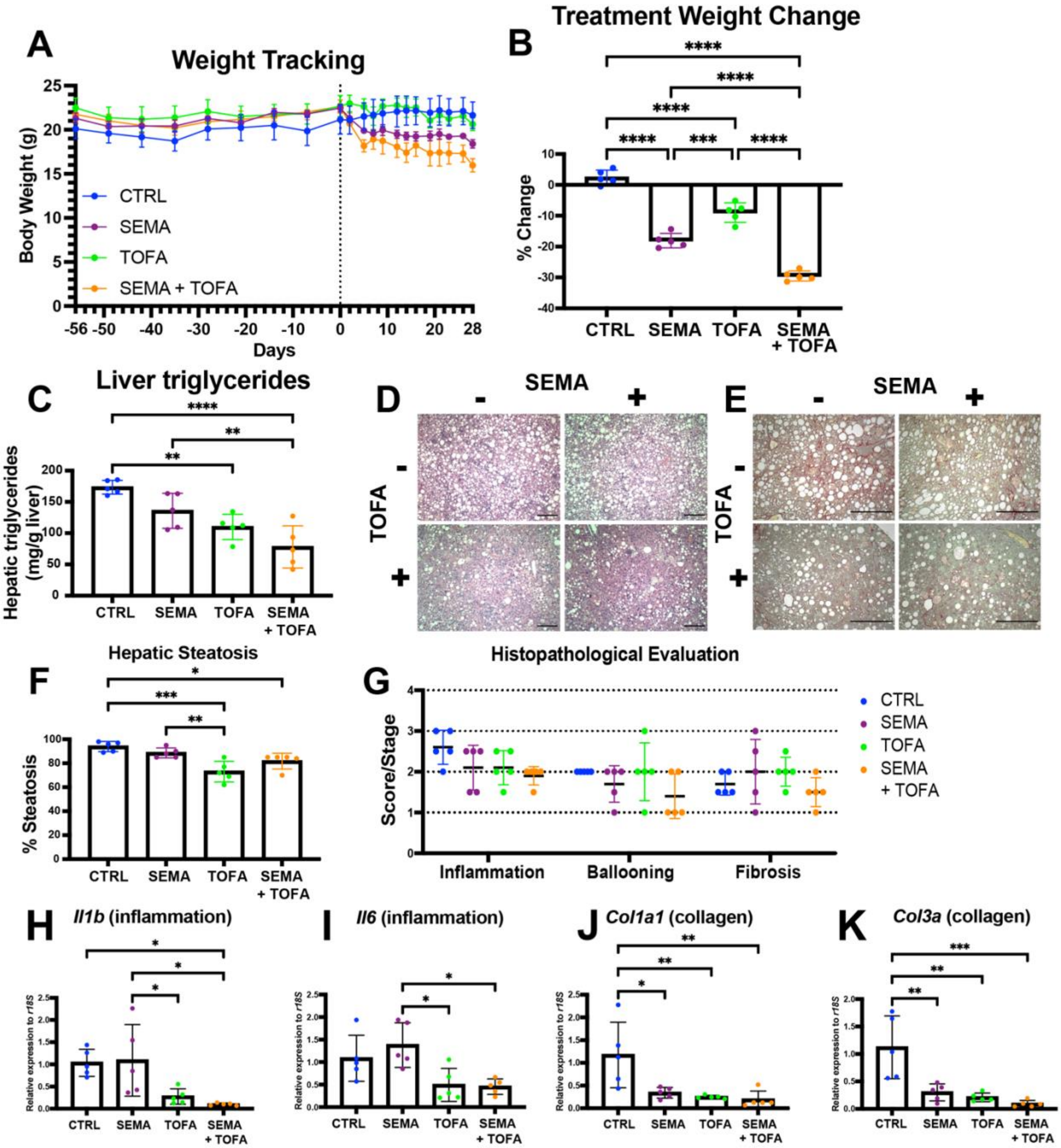

Figure S12. TOFA and semaglutide combine to address pathological features associated with MASLD/MASH progression in CDAA-HFD mouse model.

(A – K) Male C57BL/6J mice were placed on a CDAA-HFD for eight weeks and treated for four weeks with vehicle, oral TOFA gavage (250 mg/kg/day), semaglutide injection (5 nmol/kg/day), or combination of TOFA gavage and semaglutide injection; n = 5/group.

- **(A)** Body weight
- **(B)** Weight change
- **(C)** Liver triglycerides
- **(D)** Representative H&E staining of liver sections. Scale bars, 200 µM (inset).
- **(E)** Representative Sirius Red staining of liver sections. Scale bars, 200 µM (inset).
- **(F)** Hepatic steatosis

(G) MASH activity scores
(H) RT-qPCR of hepatic *Il1b* mRNA transcripts.
(I) RT-qPCR of hepatic *Il6* mRNA transcripts.
(J) RT-qPCR of hepatic *Col1a1* mRNA transcripts.
(K) RT-qPCR of hepatic *Col3a* mRNA transcripts.

Data are presented as mean $\pm$ SD. *p < 0.05, **p < 0.01, ***p < 0.001, ****p < 0.0001 (one way ANOVA).

Supplemental Table 1. Fecal gross energy content from direct calorimetry. From Supplemental Figure 4A, fecal energy content was measured by bomb calorimetry and the following reported: gross fecal energy (cal/g) and total food (HFD) energy (kcal/day) made up of total fecal energy (unabsorbed) (kcal/day) and digested energy (absorbed) (kcal/day). Rows represent individual subject replicates (n = 5/group).

	Gross Fecal Energy (cal/g)	Total Food Energy (kcal/day)	Total Fecal Energy (kcal/day)	Digested Energy (kcal/day)
Group 1 (Control)	3927.11	23.8	0.972	22.9
	4211.70	20.2	0.804	19.4
	3932.97	15.5	0.722	14.7
	3845.77	14.1	0.690	13.5
	3870.21	19.4	0.795	18.6
Control Group Average ± SD	3957.55 ± 65.67	18.6 ± 1.7	0.80 ± 0.05	17.8 ± 1.7
Group 2 (TOFA)	4766.04	14.1	0.889	13.3
	4245.95	18.3	0.940	17.4
	4081.51	17.8	0.578	17.2
	4168.38	16	0.757	15.2
	3963.21	21.5	0.866	20.6
TOFA Group Average ± SD	4245.02 ± 138.47	17.6 ± 1.2	0.81 ± 0.06	16.7 ± 1.2
Unpaired two tailed t-test (Control v. TOFA)	p=0.098(ns)	p=0.635(ns)	p=0.913(ns)	p=0.626(ns)

Supplemental Table 2. From Supplemental Figure 4C–E, descriptive statistics for ANCOVA analysis of various covariates on energy expenditure at specified temperature conditions

Temperature	Parameter	Coefficient	SE	t-value	p-value
**Room Temperature (23°C)**	Lean mass	0.0147	0.0051	2.88	*0.013
	Fat mass	-0.0018	0.0032	-0.56	0.583
	Treatment effect	0.098	0.029	3.38	**0.005
	R^2	0.621			
**Thermoneutral (30°C)**	Lean mass	0.0132	0.0043	3.07	**0.009
	Fat mass	-0.0025	0.0027	-0.93	0.371
	Treatment effect	0.050	0.018	2.78	*0.016
	R^2	0.584			
**Cold (4°C)**	Lean mass	0.0198	0.0078	2.54	*0.025
	Fat mass	-0.0011	0.0049	-0.22	0.826
	Treatment effect	0.031	0.038	0.82	0.429
	R^2	0.412			
